# Supplementary material for: Non-uniform dystrophin re-expression after CRISPR-mediated exon excision in the dystrophin/utrophin double-knockout mouse model of DMD
Source: Mol Ther Nucleic Acids. 2022 Oct 23;30:379–97. doi: 10.1016/j.omtn.2022.10.010 (PMC9664411; doi:10.1016/j.omtn.2022.10.010)
Supplement: Document S1. Figures S1–S8 and Tables S1–S4 [file mmc1.pdf]

OMTN, Volume *30*

Supplemental information

Non-uniform dystrophin re-expression after CRISPR-mediated exon excision in the dystrophin/ utrophin double-knockout mouse model of DMD

Britt Hanson, Sofia Stenler, Nina Ahlskog, Katarzyna Chwalenia, Nenad Svrzikapa, Anna M.L. Coenen-Stass, Marc S. Weinberg, Matthew J.A. Wood, and Thomas C. Roberts

Supplemental Information

Table S1

Targets for sgRNA sequences.

ID	Target Sequence (5' to 3')
**Dmd-ex23-sgRNA-1**	TTATTTACCTTCTTCTTGAT
**Dmd-ex23-sgRNA-2**	CAAATATGCGTGTTAGTGTA
**Dmd-ex23-sgRNA-3**	AGTCCTTCAAAGATATTGAT
**Dmd-ex23-sgRNA-4**	CAAAAGCCAAATCTATTTCA

Table S2

List of primer sequences used in this study.

Exon skipping RT-qPCR assay probes include 5′ terminal fluorophores (either FAM or HEX), 3′-terminal Iowa Black fluorescence quencher, and contain internal ZEN modifications. Barcode regions in the primers used for long-read amplicon sequencing are highlighted in bold.

ID	Sequence (5' to 3')
**gDNA Analysis**	
**Dmd-check-Fwd**	GCCTAAATGTCTTAATAATGTTTCAC
**Dmd-check-Rev**	GCTGTGAGCTAAATCATATCTACA
**Exon Skipping RT-qPCR**	
**qExon22-24-Fwd**	CTGAATATGAAATAATGGAGGAGAGACTCG
**qExon22-24-Rev**	CTTCAGCCATCCATTTCTGTAAGGT
**qExon22-24-Probe**	/5FAM/ATGTGATTC/ZEN/TGTAATTTCC/3IABkFQ/
**qExon23-24-Fwd**	CAGGCCATTCCTCTTTCAGG
**qExon23-24-Rev**	GAAACTTTCCTCCCAGTTGGT
**qExon23-24-Probe**	/5HEX/TCAACTTCA/ZEN/GCCATCCATTTCTGTAAGGT/3IABkFQ/
**DNA qPCR**	
**qSaCas9-Fwd**	CCAACGCCGATTTCATCTTC
**qSaCas9-Rev**	GATCTCTTTGTACTCCTGCTCG
**Long-read amplicon sequencing**	
**A701_Dmd_Check_Fwd**	**ATCACGAC**GCCTAAATGTCTTAATAATGTTTCAC
**A501_Dmd_Check_Rev**	**AAGGTTCA**GCTGTGAGCTAAATCATATCTACA
**A702_Dmd_Check_Fwd**	**ACAGTGGT**GCCTAAATGTCTTAATAATGTTTCAC
**A502_Dmd_Check_Rev**	**ACTTAGCA**GCTGTGAGCTAAATCATATCTACA
**A703_Dmd_Check_Fwd**	**CAGATCCA**GCCTAAATGTCTTAATAATGTTTCAC
**A503_Dmd_Check_Rev**	**AGAGAACA**GCTGTGAGCTAAATCATATCTACA
**A704_Dmd_Check_Fwd**	**ACAAACGG**GCCTAAATGTCTTAATAATGTTTCAC
**A504_Dmd_Check_Rev**	**GTGTCTTA**GCTGTGAGCTAAATCATATCTACA
**A705_Dmd_Check_Fwd**	**ACCCAGCA**GCCTAAATGTCTTAATAATGTTTCAC
**A505_Dmd_Check_Rev**	**TCGATTAG**GCTGTGAGCTAAATCATATCTACA

Table S3

List of TaqMan assays used in this study.

All products were obtained from Thermo Fisher Scientific.

Target	Product ID	Detection Channel
**Small RNA TaqMan RT-qPCR**		
**mmu-miR-1a-3p**	002222	FAM
**mmu-miR-133a-3p**	002246	FAM
**mmu-miR-206-3p**	000510	FAM
**mmu-miR-483-3p**	002560	FAM
**cel-miR-39**	000200	FAM
**DNA qPCR**		
Actb	Mm00607939_s1*	VIC

Table S4

Antibodies used in this study.

Target protein	Host	Product ID	Manufacturer	Dilution
**Western Blot**				
**Dystrophin (DMD)**	Mouse mAb	NCL-DYS1	Leica Biosystems	1:100
**Vinculin (VCL)**	Mouse mAb	V9131	Sigma-Aldrich/Merck	1:200
**Anti-mouse IgG, HRP-linked**	Horse	7076	Cell Signaling Technology	1:5,000
**Immunofluorescence**				
**Dystrophin (DMD)**	Rabbit pAb	ab15277	Abcam	1:1,000
**β-Dystroglycan (DAG1)**	Mouse mAb	NCL-b-DG	Leica Biosystems	1:100
**α-Dystrobrevin (DTNA1)**	Rabbit pAb	Alpha-1CTFP	In house (gift from K.E. Davies)	1:100
**nNOS (NOS1)**	Rabbit mAb	Ab76067	Abcam	1:100
**Laminin subunit alpha 2 (LAMA2)**	Rat mAb	L0663 (clone: 4H8-2)	Sigma-Aldrich/Merck	1:1,000
**Anti-rabbit IgG Alexa Fluor 594**	Goat	ab150080	Abcam	1:500
**Anti-rat IgG Alexa Fluor 488**	Goat	ab150157	Abcam	1:500

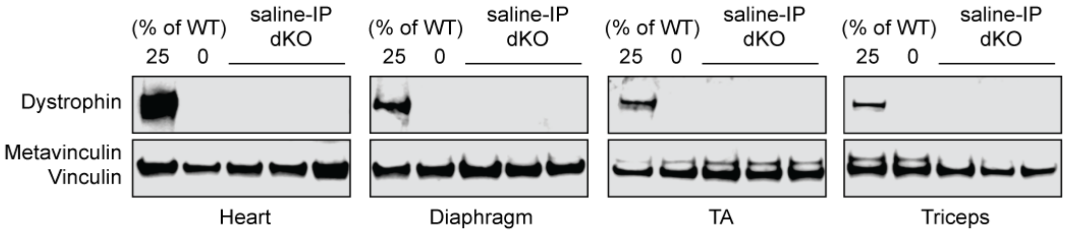

Figure S1

Dystrophin protein is undetectable by western blot in saline-treated dKO mouse tissues.

Western blot for dystrophin in 20 μg of total protein obtained from the heart, diaphragm, TA, and triceps muscles. Positive control samples contain a mixture of 25% WT (C57/BL10) with 75% dystrophin-deficient *mdx* protein. Negative control samples contain *mdx* protein only. The remaining samples consist of dKO animals treated with saline by IP injection. Vinculin was included as loading control.

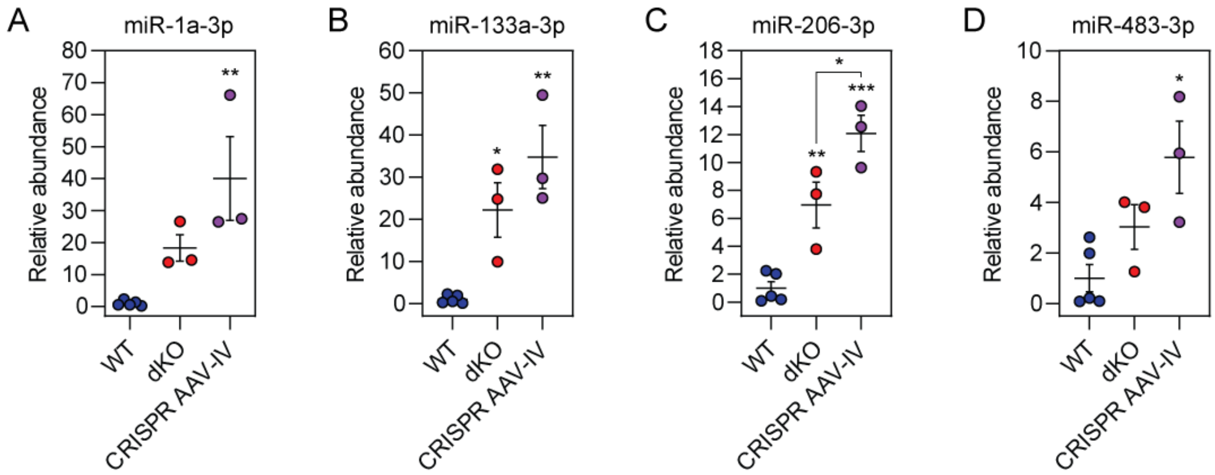

Figure S2

miRNA biomarkers are not restored to wild-type levels in CRISPR AAV-treated dKO mouse serum.

dKO mice were treated by intravenous (IV) injection via the facial vein at postnatal day two (P2) with 1×10^{11} vg of SaCas9-AAV and 5×10^{11} vg of dual sgRNA-AAV and sacrificed at the humane end point. Serum was harvested from the animals immediately postmortem (n=3) and RNA extracted. Serum RNA was analyzed for (**A**) miR-1a-3p, (**B**) miR-133a-3p, (**C**) miR-206-3p, and (**D**) miR-483-3p by small RNA TaqMan RT-qPCR. Serum from un-injected wild-type (WT) (n=5) and dKO (n=3) animals served as controls (10-weeks-old). Values are mean±SEM. Statistically significant differences were tested by one-way ANOVA with Bonferroni *post hoc* test, *P<0.05, **P<0.01, ***P<0.001.

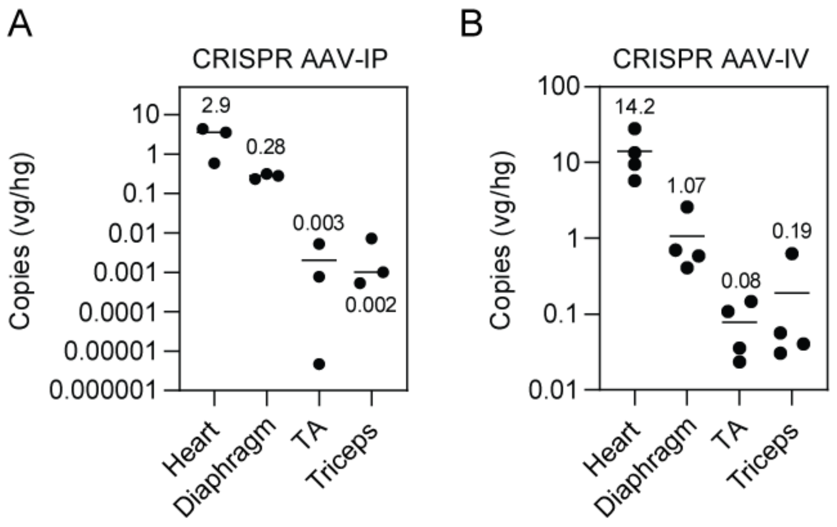

Figure S3

Biodistribution of SaCas9 vector genomes.

DNA was extracted from CRISPR AAV-treated dKO mice for (**A**) intraperitoneal (IP, *n*=3), and (**B**) intravenous (IV, *n*=4) routes of administration. Absolute quantification qPCR was used to detect SaCas9-AAV vector genome (vg) copies using primers against the SaCas9 transgene and data normalized to the copy number of host genomes (hg) measured using qPCR primers for *Actb*. Mean copy numbers are indicated by horizontal bars, and the mean value shown next to each column.

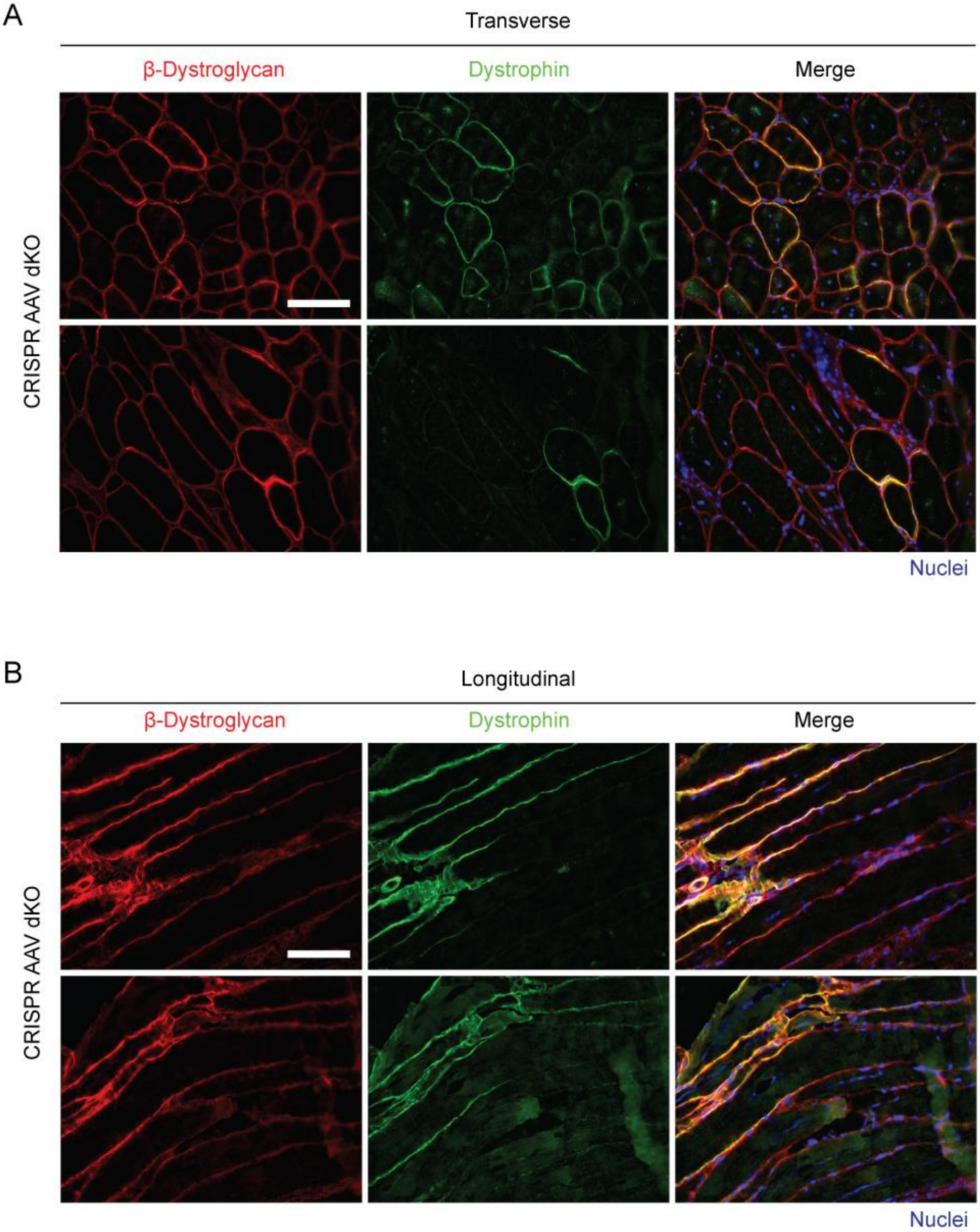
A
Transverse
β-Dystroglycan
Dystrophin
Merge
CRISPR AAV dKO
Nuclei
B
Longitudinal
β-Dystroglycan
Dystrophin
Merge
CRISPR AAV dKO
Nuclei

Figure S4

β-dystroglycan is expressed in a within-fiber patchy manner, and is colocalized with dystrophin in dKO muscle after CRISPR AAV treatment.

Representative β-dystroglycan IF staining in (**A**) transverse and (**B**) longitudinal TA muscle sections of dKO mice treated with CRISPR AAV9 particles. Sections were co-stained for dystrophin and nuclei stained with DAPI. Images were taken at 20× magnification, and scale bars represent 100 μm.

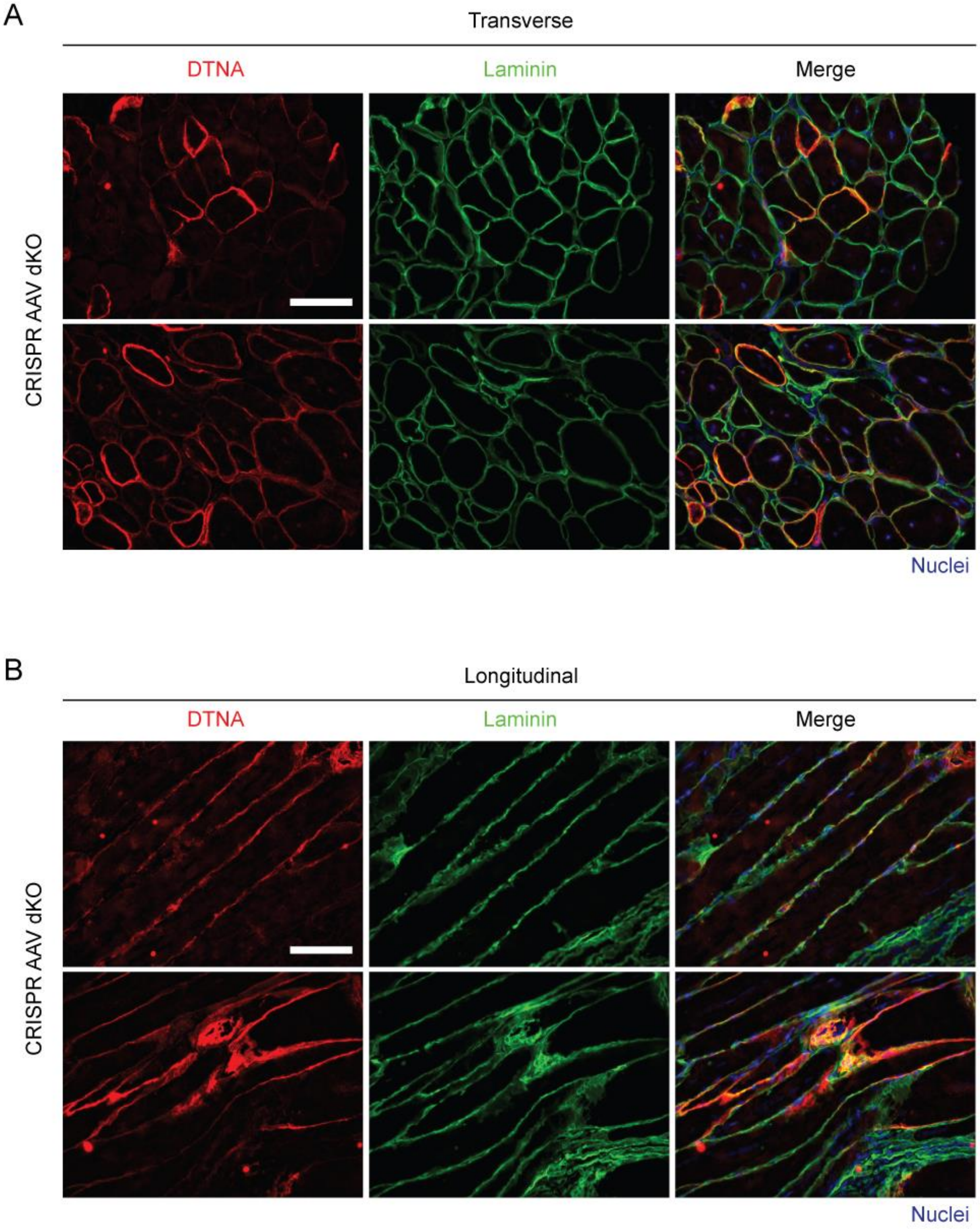
A
Transverse
DTNA
Laminin
Merge
CRISPR AAV dKO
Nuclei
B
Longitudinal
DTNA
Laminin
Merge
CRISPR AAV dKO
Nuclei

Figure S5

DTNA protein is re-expressed in a within-fiber patchy manner in dKO muscle after CRISPR AAV treatment.

Representative DTNA (α-dystrobrevin) IF staining in (**A**) transverse and (**B**) longitudinal TA muscle sections of dKO mice treated with CRISPR AAV9 particles. Sections were co-stained for laminin to delineate myofiber boundaries and nuclei stained with DAPI. Images were taken at 20× magnification, and scale bars represent 100 μm.

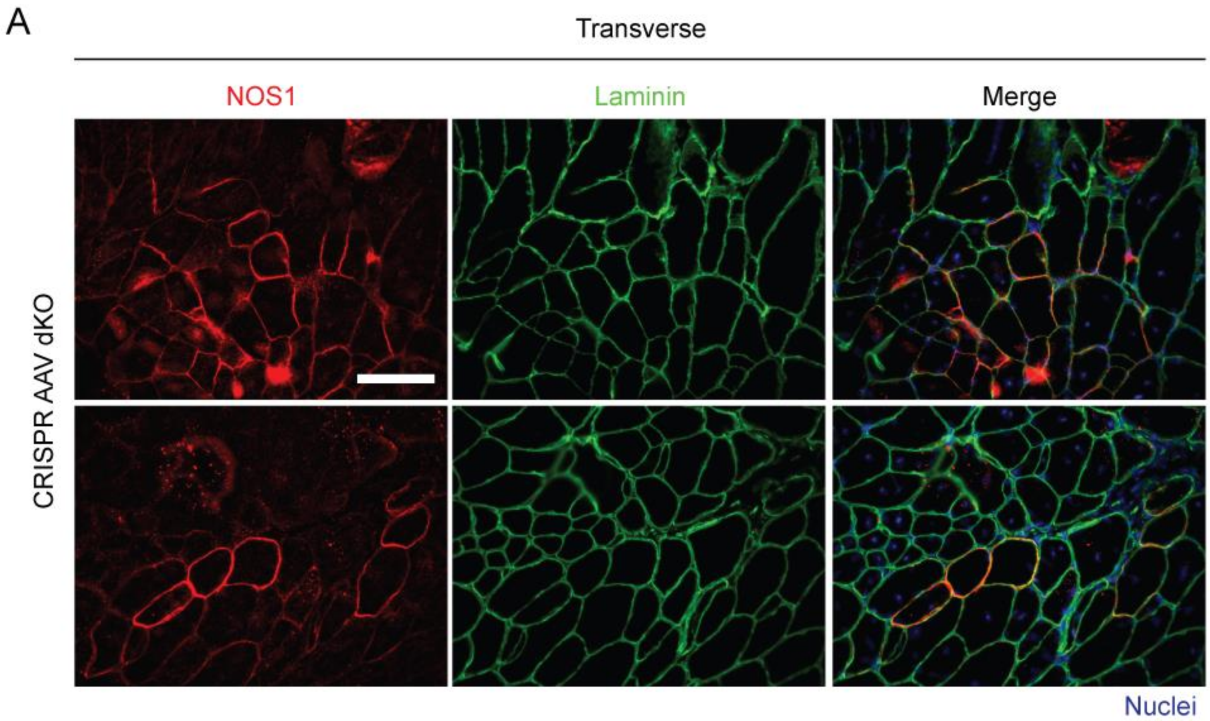
A
Transverse
NOS1
Laminin
Merge
CRISPR AAV dKO
Nuclei

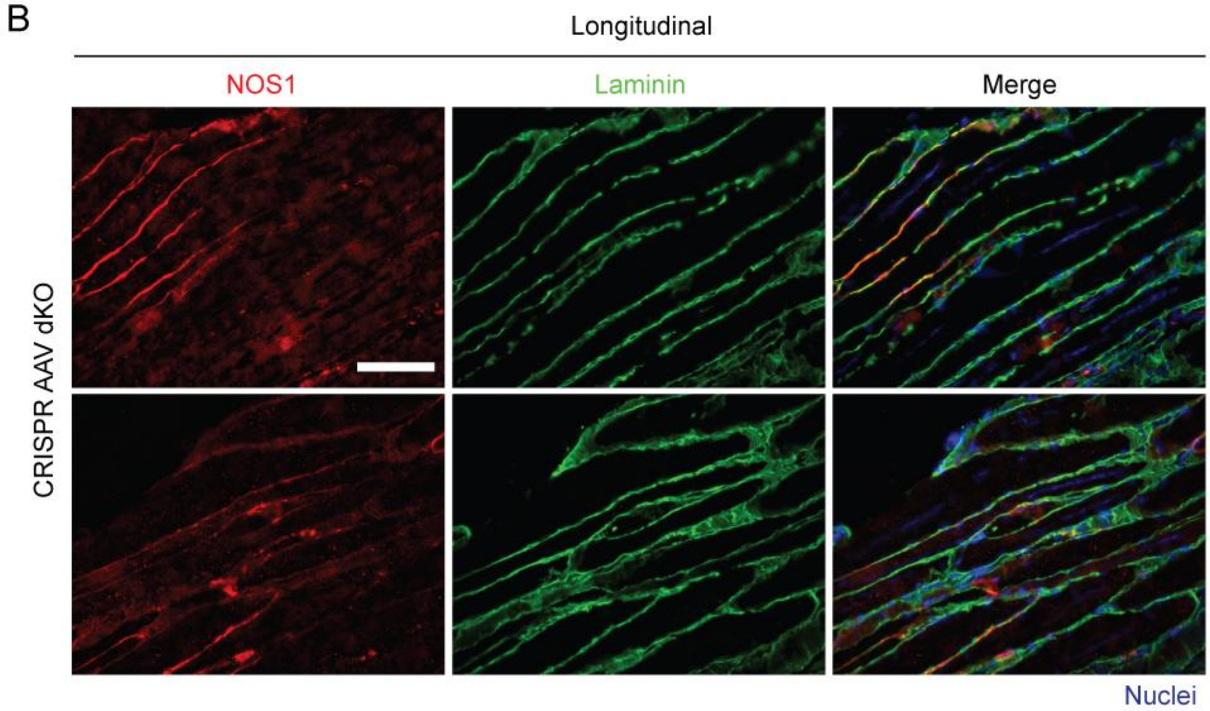
B
Longitudinal
NOS1
Laminin
Merge
CRISPR AAV dKO
Nuclei

Figure S6

NOS1 protein is expressed in a within-fiber patchy manner in dKO muscle after CRISPR AAV treatment.

Representative NOS1 (neuronal nitric oxide synthase, nNOS) IF staining in (**A**) transverse and (**B**) longitudinal TA muscle sections of dKO mice treated with CRISPR AAV9 particles. Sections were co-stained for laminin to delineate myofiber boundaries and nuclei stained with DAPI. Images were taken at 20× magnification, and scale bars represent 100 μm.

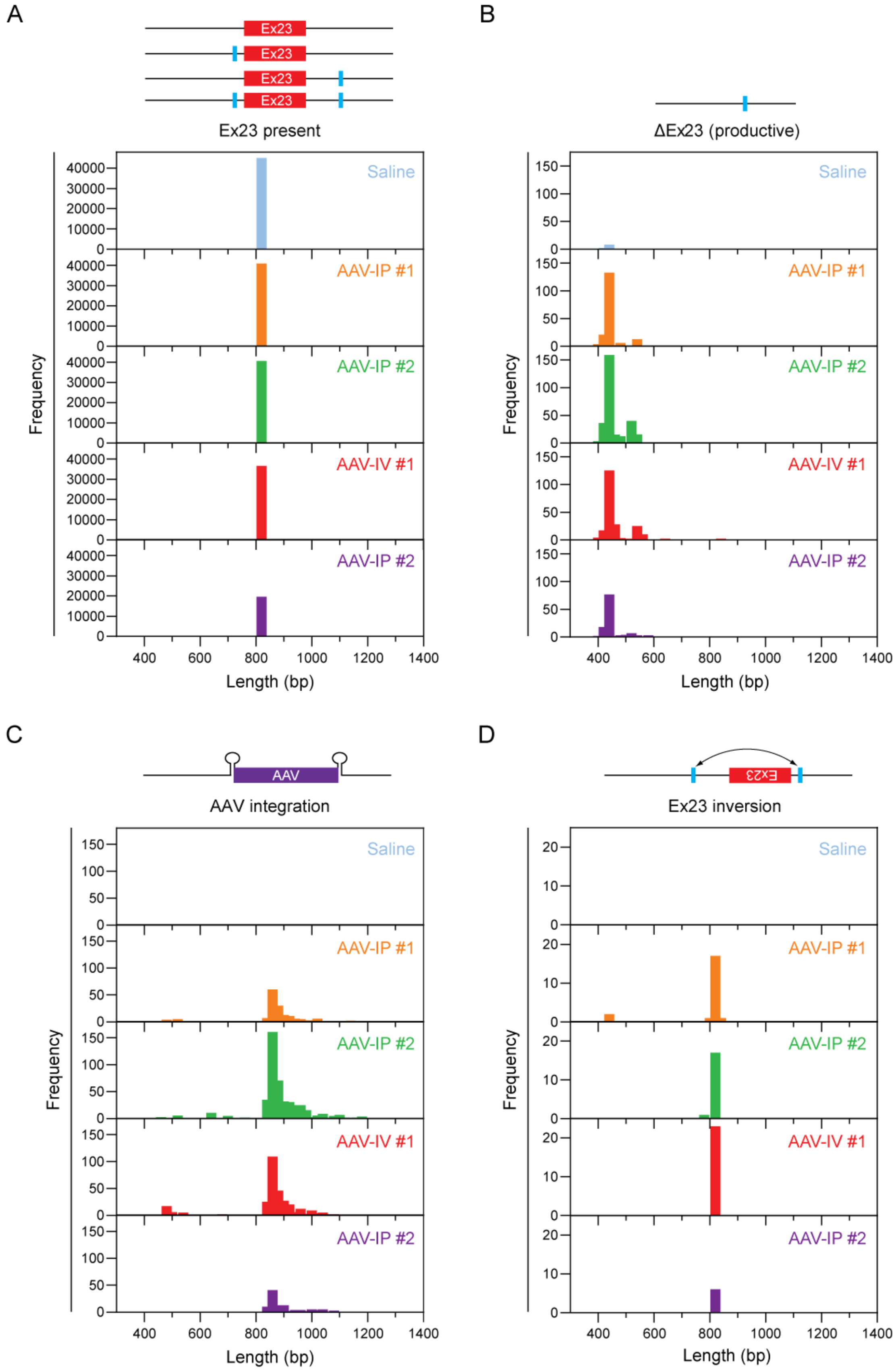
A
Ex23
Ex23
Ex23
Ex23
Ex23 present
Frequency
Saline
AAV-IP #1
AAV-IP #2
AAV-IV #1
AAV-IP #2
Length (bp)
B
ΔEx23 (productive)
Frequency
Saline
AAV-IP #1
AAV-IP #2
AAV-IV #1
AAV-IP #2
Length (bp)
C
AAV
AAV integration
Frequency
Saline
AAV-IP #1
AAV-IP #2
AAV-IV #1
AAV-IP #2
Length (bp)
D
Ex23
Ex23 inversion
Frequency
Saline
AAV-IP #1
AAV-IP #2
AAV-IV #1
AAV-IP #2
Length (bp)

Figure S7

Size distributions for read classification groups following long-read amplicon sequencing. Sequenced amplicons from CRISPR-treated dKO heart samples, or saline control, were assigned to separate categories (**Figure 8**) and read length size distributions determined for (**A**) unedited sequence/indels present at the sgRNA cut site(s), (**B**) ΔEx23 (productive editing), (**C**) presence of integrated AAV-derived sequences, and (**D**) *Dmd* Ex23 present in the reverse orientation (i.e. Ex23 inversion). Schematic representations of possible amplicons are shown for each category.

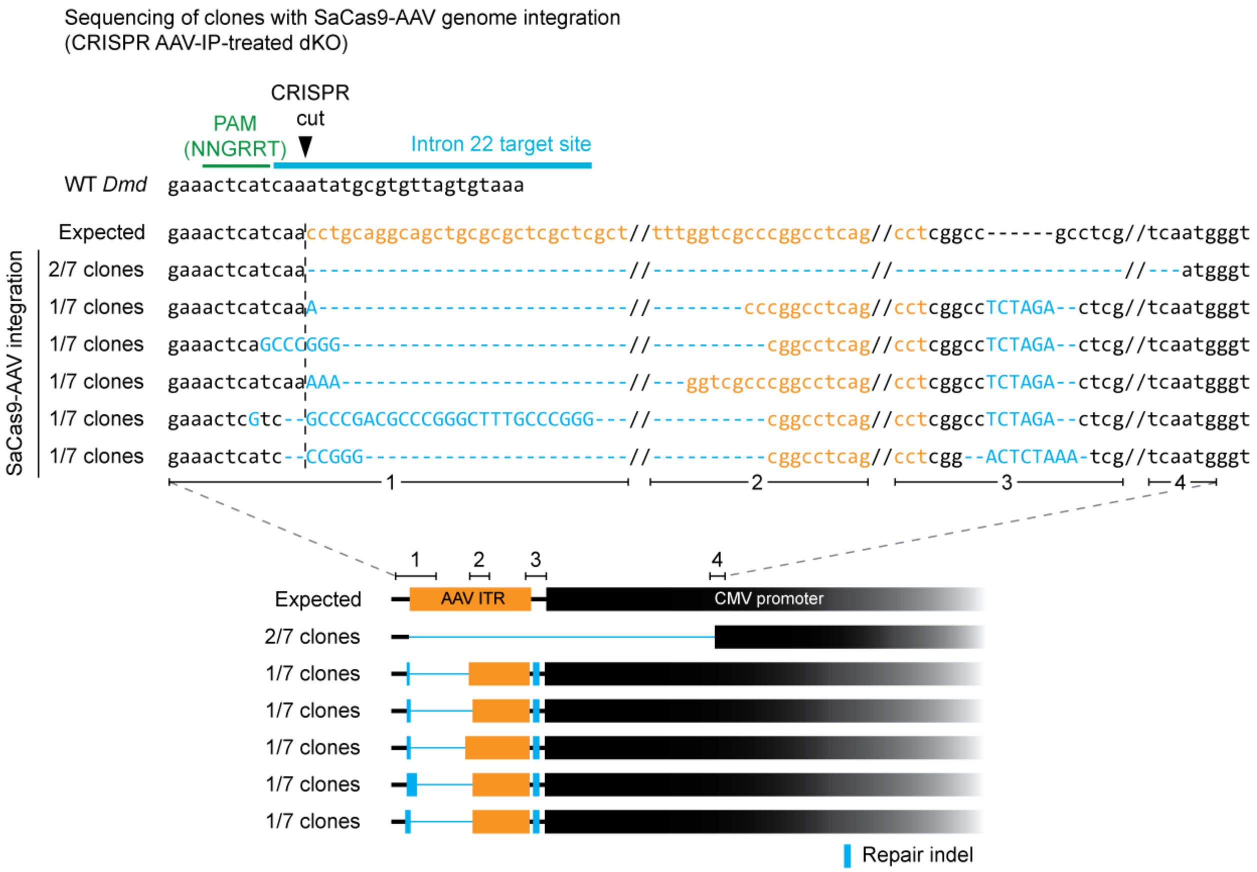

Figure S8

Detection of SaCas9-AAV vector backbone at the 5′ CRISPR cut site by cloning and Sanger sequencing.

PCR was carried out on DNA harvested from the heart tissue of dKO mice treated via intraperitoneal (IP) or intravenous (IV) injection of CRISPR AAVs with primers specific to *Dmd* intron 22 and the CMV promoter of the SaCas9-AAV genome (**Figure 8D**). Amplicons obtained from one AAV-IP treated animal were cloned into shuttle vectors and seven single clones analyzed by Sanger sequencing. Fragments of the SaCas9-AAV vector backbone was detected in all clones tested. Uppercase nucleotides indicate insertions of random bases not present in the WT sequence.
