# Supplementary material for: Non-uniform dystrophin re-expression after CRISPR-mediated exon excision in the dystrophin/utrophin double-knockout mouse model of DMD
Source: Mol Ther Nucleic Acids. 2022 Oct 23;30:379–97. doi: 10.1016/j.omtn.2022.10.010 (PMC9664411; doi:10.1016/j.omtn.2022.10.010)
Supplement: Document S2. Article plus supplemental information [file mmc2.pdf]

Molecular Therapy
Nucleic Acids
Original Article

Non-uniform dystrophin re-expression after CRISPR-mediated exon excision in the dystrophin/utrophin double-knockout mouse model of DMD

Britt Hanson,[1,2] Sofia Stenler,[2,8] Nina Ahlskog,[1,3] Katarzyna Chwalenia,[1,3] Nenad Svrzikapa,[1,3,4] Anna M.L. Coenen-Stass,[2,9] Marc S. Weinberg,[5,6] Matthew J.A. Wood,[1,3,7] and Thomas C. Roberts[1,3,7]

[1]Department of Paediatrics, University of Oxford, South Parks Road, Oxford OX1 3QX, UK; [2]Department of Physiology, Anatomy and Genetics, University of Oxford, South Parks Road, Oxford OX1 3QX, UK; [3]Institute of Developmental and Regenerative Medicine, University of Oxford, IMS-Tetsuya Nakamura Building, Old Road Campus, Roosevelt Dr, Headington, Oxford OX3 7TY, UK; [4]Wave Life Sciences Ltd., Cambridge, MA 02138, USA; [5]Antiviral Gene Therapy Research Unit, Department of Molecular Medicine and Haematology, University of the Witwatersrand Medical School, WITS 2050, South Africa; [6]Asklepios BioPharmaceutical, Inc., Research Triangle Park, NC 27709, USA; [7]MDUK Oxford Neuromuscular Centre, South Parks Road, Oxford OX1 3QX, UK

Duchenne muscular dystrophy (DMD) is the most prevalent inherited myopathy affecting children, caused by genetic loss of the gene encoding the dystrophin protein. Here we have investigated the use of the *Staphylococcus aureus* CRISPR-Cas9 system and a double-cut strategy, delivered using a pair of adeno-associated virus serotype 9 (AAV9) vectors, for dystrophin restoration in the severely affected dystrophin/utrophin double-knockout (dKO) mouse. Single guide RNAs were designed to excise *Dmd* exon 23, with flanking intronic regions repaired by non-homologous end joining. Exon 23 deletion was confirmed at the DNA level by PCR and Sanger sequencing, and at the RNA level by RT-qPCR. Restoration of dystrophin protein expression was demonstrated by western blot and immunofluorescence staining in mice treated via either intraperitoneal or intravenous routes of delivery. Dystrophin restoration was most effective in the diaphragm, where a maximum of 5.7% of wild-type dystrophin expression was observed. CRISPR treatment was insufficient to extend lifespan in the dKO mouse, and dystrophin was expressed in a within-fiber patchy manner in skeletal muscle tissues. Further analysis revealed a plethora of non-productive DNA repair events, including AAV genome integration at the CRISPR cut sites. This study highlights potential challenges for the successful development of CRISPR therapies in the context of DMD.

INTRODUCTION

Mutations in the gene encoding the dystrophin protein (*DMD*) lead to Duchenne muscular dystrophy (DMD), where dystrophin protein is effectively absent, and Becker muscular dystrophy (BMD), where in-frame deletions typically result in production of a partially truncated dystrophin protein. DMD is a lethal, currently incurable, progressive muscle-wasting condition and the most common inherited myopathy affecting children.[1] Loss of dystrophin leads to myofiber fragility, cycles of muscle turnover, persistent inflammation, and fibro/fatty muscle degeneration.[2–4] DMD patients suffer from progressive muscle wasting and typically become wheelchair bound around the age of 12 years.[5] While improvements in disease management have extended lifespan, patients typically still succumb to cardiac or respiratory failure around the age of 30 years.[6–8] In contrast, disease severity in BMD patients varies widely, although it is generally considered to be much less severe than DMD, with a later onset of symptoms.[9] In some rare cases, BMD patients are effectively asymptomatic, despite large internal deletions within the *DMD* gene.[10–12] The molecular reason for the differences in pathological severity between these dystrophinopathies relates to the nature of their respective causative mutations.[13] In DMD, the dystrophin translation reading frame is typically disrupted, often by whole-exon deletions. In the case of BMD, the dystrophin mutation often does not affect the translation reading frame, and so some degree of dystrophin functionality is retained.[14]

The *DMD* gene consists of 79 exons, many of which encode for redundant structural domains that are not essential for dystrophin function.[15] DMD is therefore amenable to splice correction therapy in which short antisense oligonucleotides targeting splicing motifs in the dystrophin pre-mRNA effectively mask specific exons from the splicing machinery, leading to their exclusion from the processed mRNA (i.e., exon skipping).[16] This alternative splicing event restores the translation reading frame and rescues expression of a truncated, and partially functional, pseudo-dystrophin. The aim of exon

Received 12 May 2022; accepted 20 October 2022;
https://doi.org/10.1016/j.omtn.2022.10.010.

[8]Present address: Uppsala University, Office for Science and Technology, SE-751 05 Uppsala, Sweden

[9]Present address: Merck KGaA, Frankfurter Str. 250, 64293 Darmstadt, Germany

Correspondence: Dr Thomas C. Roberts, Institute of Developmental and Regenerative Medicine, University of Oxford, IMS-Tetsuya Nakamura Building, Old Road Campus, Roosevelt Dr, Headington, Oxford, OX3 7TY, UK.
E-mail: thomas.roberts@idrm.ox.ac.uk

This is an open access article under the CC BY license (http://creativecommons.org/licenses/by/4.0/).

skipping is therefore to convert the DMD phenotype to a milder BMD-like phenotype. To date, there are four US Food and Drug Administration (FDA)-approved exon-skipping drugs (eteplirsen, golodirsen, viltolarsen, and casimersen).[17] These therapies are currently very expensive (~US$300,000 per patient *per annum*), show limited efficacy (i.e., restoration of ~1% of normal dystrophin protein levels for eteplirsen),[18] and require lifelong administration. There is therefore intense interest in more permanent dystrophin restoration strategies, such as gene editing.

In recent years, the gene editing field has experienced a revolution with the discovery and development of the clustered regularly interspaced short palindromic repeats-CRISPR associated protein 9 (CRISPR-Cas9) system,[19–21] for which the Nobel Prize in Chemistry was awarded to Doudna and Charpentier in 2020. In the most commonly used configuration, the CRISPR approach requires two components: (1) the Cas9 enzyme, which induces double-strand breaks (DSBs) in genomic DNA (gDNA); and (2) a single guide RNA (sgRNA) that specifies the target DNA sequence. The use of an RNA component makes this technology highly versatile as there are relatively few constraints on what sequence can be targeted, and sgRNAs can be generated rapidly. Furthermore, the CRISPR-Cas9 system enables facile multiplexing of genome editing events, such that multiple loci can be targeted simultaneously.[20] CRISPR-induced DSB lesions are resolved by cellular DNA damage repair pathways, the most common being the error-prone non-homologous end joining (NHEJ) pathway, and homology directed repair (HDR). CRISPR-based approaches for DMD have mainly focused on strategies relying on the NHEJ DNA repair pathway, as HDR is generally not active in quiescent or terminally differentiated cells.[22] The leading approaches are (1) a double-cut strategy in which a target exon(s) is excised, essentially achieving permanent exon skipping,[23–28] and (2) a single-cut strategy aimed at inducing an indel for the purposes of either disrupting a splice signal or reframing an exon.[29–34] *In vivo* delivery of the Cas9 and sgRNA transgenes is typically achieved with the use of adeno-associated virus (AAV) vectors,[35] of which there are several muscle-tropic serotypes.[36,37] AAVs have been used extensively for gene therapy applications and have already demonstrated safe and efficacious delivery in clinical trials.[38,39] Notably, the commonly used *Streptococcus pyogenes* Cas9 (SpCas9) protein is too large to be effectively packaged into AAV vectors, which has led to the development of smaller orthogonal Cas9 proteins.[40] The compact (3.2 kb) Cas9 derived from *Staphylococcus aureus* (i.e., SaCas9) has emerged as the most widely utilized variant of choice.[41]

The most commonly used mouse model of DMD is the *mdx* mouse, which recapitulates some aspects of DMD pathology. Specifically, a "crisis" period of extensive myonecrosis and muscle regeneration is observed between 2 and 5 weeks of age,[42] with more severe histopathological features reminiscent of DMD pathology (i.e., fibro/fatty degeneration) observed much later (~80 weeks).[43] Cardiomyopathy is generally not observed in the *mdx* mouse unless experimentally induced.[44] Importantly, the *mdx* mouse exhibits only a small decrease in lifespan, and impairment of muscle function is relatively mild. Disease in the *mdx* mouse is similar to that observed in other dystrophin-null models.[45] In contrast, the dKO mouse (a double knockout of dystrophin and its paralog utrophin, *Utrn*) exhibits much more severe pathology, including kyphosis, respiratory difficulties, and premature death (animals typically die ~8–10 weeks of age).[46–49] Notably, exon-skipping therapy to rescue dystrophin protein expression in the dKO mouse reverses dystrophic pathology and dramatically extends lifespan.[48,49] Importantly, the dKO mouse carries an identical genetic mutation to that of the *mdx* mouse (i.e., a nonsense mutation in *Dmd* exon 23) and so these two strains can be treated using common exon-skipping or CRISPR-based therapeutic strategies.

Here, we report *Dmd* gene editing and dystrophin restoration in the severely affected dKO mouse model of DMD using an AAV-delivered (two vector), double-cut CRISPR strategy. However, the observed levels of gene editing were insufficient to extend lifespan in the dKO mouse, or to correct circulating microRNA (miRNA) biomarker levels. Dystrophin protein expression in the treated animals was localized to the sarcolemma but exhibited a within-fiber patchy distribution pattern. Long-read sequencing of the editing target site revealed a plethora of non-productive editing outcomes, including AAV backbone integration events and indels that disrupt the sgRNA target sites. We propose that inefficient editing using this approach will inevitably lead to the generation of chimeric fibers containing both dystrophin-expressing and non-dystrophin-expressing myonuclei, and that the resulting incomplete pattern of sarcolemmal dystrophin may be insufficient to correct the dystrophic phenotype.

RESULTS

Screening of *Dmd*-targeting guide RNAs in murine fibroblasts

In order to correct the genetic defect in the dKO mouse, we have utilized a multiplex genome editing strategy intended to excise *Dmd* exon 23, similar to approaches described previously.[23–25,27,31] This approach aims to induce DNA DSBs on either side of the target exon with the resulting genetic lesion repaired via the NHEJ pathway. The expected productive editing outcome is the excision of exon 23 and the fusion of the flanking introns (Figure 1A). Putative SaCas9 protospacer adjacent motif (PAM) sites (i.e., NNGRRT,[41] where R = A or G) were identified in the intronic sequence surrounding exon 23. Four potential target sites were selected (two each on either side of the exon) and sgRNAs cloned into U6 expression cassettes (Figure 1B). To identify the optimal dual guide combination, mouse fibroblast cells (10T1/2) were transfected with SaCas9 and all four possible combinations of the sgRNA plasmids (Figure 1C). Genome editing was assessed by PCR using primers that span the edit site and produce an 833 bp amplicon. Excision of *Dmd* exon 23 was observed for two of the four combinations, as indicated by the detection of a smaller PCR product of ~446 bp. The optimal combination of sgRNAs (2 + 4) exhibited gene editing efficiency of ~4.7% and was selected for further development. The edited amplicon was analyzed by direct Sanger sequencing to characterize NHEJ-induced indels (Figure 1D). The major product in the sequencing chromatogram corresponded to seamless repair of the up- and downstream introns

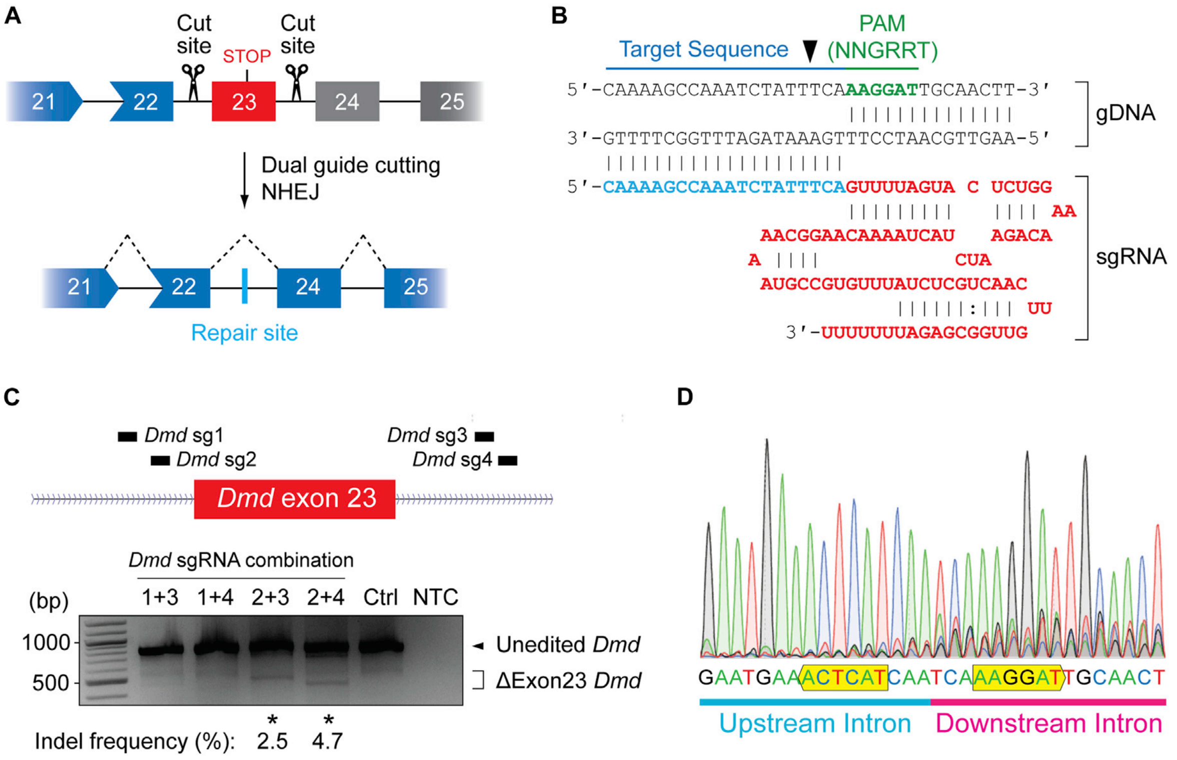

Figure 1. CRISPR-Cas9-mediated *Dmd* exon 23 excision in cell culture

(A) Schematic of dual sgRNA CRISPR-Cas9-mediated excision of murine *Dmd* disease-causing exon 23 (in red), resulting in NHEJ and a repair indel within the intron that restores the *Dmd* translation reading frame. Out-of-frame exons are indicated in gray. (B) Schematic of the structure of an SaCas9 sgRNA in complex with its cognate gDNA target site. The SaCas9-specific protospacer adjacent motif (PAM) sequence (NNGRRT, where R is A or G) is shown in green and is located on the non-targeted DNA strand. (C) Screening of the activity of four combinations of sgRNAs for *Dmd* exon 23 excision in murine 10T1/2 fibroblast cells. The genomic target site was amplified by PCR and the products resolved by agarose gel electrophoresis. Successful editing is indicated by a shorter ΔExon23 *Dmd* product and editing percentages indicated for successful sgRNA combinations. (D) Sanger sequencing chromatogram confirming successful exon 23 excision and fusion of the up- and downstream intronic regions following treatment with SaCas9 sgRNA combination 2 + 4.

at the expected sgRNA cut sites (i.e., with no indel), although an increase in background signal was observed around the repair site, indicative of low level of indel formation.

CRISPR-mediated dystrophin rescue in the dKO mouse after intraperitoneal injection

To evaluate the potential for CRISPR-Cas9-mediated *Dmd* editing in the severely affected double knockout for dystrophin and utrophin (dKO) mouse *in vivo*, two separate AAV9 vectors were designed. The first vector encodes for an SaCas9 transgene with compact regulatory sequences to minimize the size of the AAV genome (i.e., the cytomegalovirus [CMV] promoter and SV40 minimal poly(A) signal). The total size of the resulting AAV genome (including inverted terminal repeats [ITRs]) was 4,806 bp, which is below the maximum limit for AAV packaging (estimated at 5.2 kb).[50] For the second AAV vector, two different RNA polymerase III promoters (the human H1 and U6 small nuclear RNA [snRNA] promoters) were selected to drive expression of the *Dmd*-targeting sgRNAs in order to minimize the repetitive sequence within the AAV genome (Figure 2A).

Postnatal day 2 (P2) dKO mice were treated with 1×10^{11} vector genomes (vg) of SaCas9-AAV and 5×10^{11} vg of dual sgRNA-AAV, by intraperitoneal (IP) injection (Figure 2B). Treated mice were monitored until they reached the humane endpoint, at which time the heart, diaphragm, tibialis anterior (TA), and triceps were harvested in order to determine the efficacy of gene editing at the gDNA, RNA, and protein levels.

The ΔExon23 *Dmd* gene product, indicative of productive editing at the genomic target site, was detected sporadically across the tissues analyzed by PCR (Figure 2C), and successful excision and repair of the flanking intronic regions at the cut sites was confirmed by Sanger sequencing of the bulk edited PCR amplicon (Figure 2D). The sequencing chromatogram demonstrated seamless fusion of the flanking intronic regions, with an increase in background signal around the repair site indicative of low levels of indel formation. Cloning of the edited amplicon and sequencing of seven individual clones revealed three major editing events: seamless repair (2/7), a 4-bp deletion downstream of the fusion site (2/7), and an 11-bp deletion upstream of the fusion site (3/7) (Figure 2D).

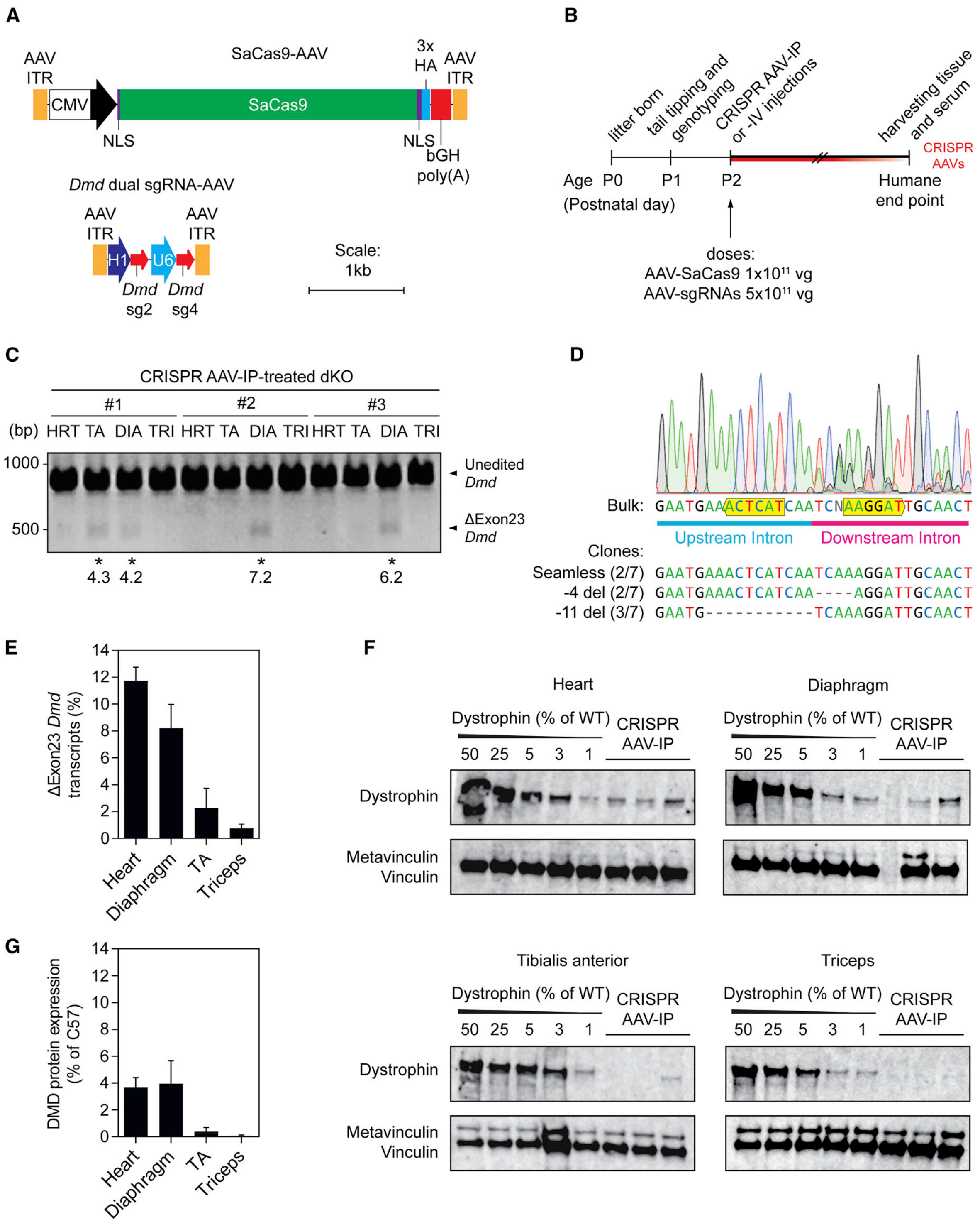

Figure 2. Evaluation of *Dmd* exon 23 excision and dystrophin restoration in dKO mice after IP injection of neonates

(A) Schematics of the SaCas9-AAV and *Dmd* dual sgRNA-AAV genomes contained within AAV2 inverted terminal repeats (ITRs). The SaCas9 gene is driven by a CMV promoter and enhancer, is flanked by an SV40 nuclear localization signal (NLS) on the N terminus and a nucleoplasmin NLS on the C terminus, is tagged with three hemagglutinin (HA) tags, and is terminated with a bGH poly(A) signal. Expression of *Dmd* sg2 and sg4 is driven by an H1 and a U6 promoter, respectively (sgRNAs selected based on results in Figure 1). (B) Treatment timeline for dKO mice with CRISPR AAV9 particles. Mice were treated by IP injection (n = 3) at postnatal day 2 (P2) with 1×10^{11} vg

(legend continued on next page)

Successful excision of the gDNA encoding *Dmd* exon 23 is expected to manifest as permanent exon skipping. As such, levels of ΔExon23 *Dmd* mRNA transcripts were determined by RT-qPCR using primers spanning exons 22–24 (i.e., exon skipped) and spanning exons 23–24 (i.e., exon unskipped). Successful exon excision was detected in all tissues tested, thereby indicating that this method is more sensitive for detecting editing than endpoint PCR analysis of gDNA. ΔExon23 *Dmd* transcript levels were highest in the heart and diaphragm at ∼12% and 8% respectively (Figure 2E). Editing in the TA and triceps was less successful, with only ∼2% and 1% ΔExon23 *Dmd* transcripts detected respectively.

A similar pattern was observed at the protein level, whereby dystrophin protein expression was observed in the heart, diaphragm, and TA muscles of the treated dKO mice, but at negligible levels in the triceps as determined by western blot (Figures 2F and 2G). The size of the restored dystrophin protein was similar to that observed in wild-type (WT) controls, which is to be expected as deletion of exon 23 results in a small (71 amino acids, 420 Da) change in the size of the dystrophin protein. Dystrophin was undetectable in saline-treated dKO control animals (Figure S1).

Immunofluorescence (IF) staining for dystrophin protein was performed to visualize the pattern of successful gene editing within CRISPR AAV-treated muscle tissue sections. Transverse sections obtained from each of the four muscle tissues were stained with anti-dystrophin fluorescent antibodies to assess the distribution of restored fibers throughout the selected tissues. Sections were co-stained for laminin (LAMA2), a basal lamina protein that delineates the boundaries of individual myofibers (Figure 3). Tissue sections from WT and saline-treated dKO mice were included as positive and negative controls, respectively. Expression of dystrophin, localized at the sarcolemma, was confirmed in all four tissues analyzed following CRISPR AAV treatment via IP injection, whereas dystrophin staining was largely absent in sections from all the muscle tissues of saline-treated dKO mice (aside from sporadic dystrophin-positive revertant fibers). In the heart and diaphragm, where the highest levels of editing were observed by RT-qPCR and western blot (Figures 2E–2G), the restored fibers were distributed throughout the tissue section. In the two limb muscles, however, the corrected fibers formed a more clustered pattern, with patches of dystrophin-positive and -negative fibers. This between-fiber patchiness may be due to mosaicism of successful gene editing early in the muscle development of these mice or may be a consequence of differences in vascular permeability throughout the muscle tissue. Regions of dystrophin-negative staining within CRISPR-corrected dystrophin-positive myofibers were also observed (Figure 3, arrowheads), which was most apparent in the TA and triceps muscles. Notably, we observed IF signal even when dystrophin levels were very low as determined by western blot, consistent with the former technique having higher sensitivity, as has been suggested previously.[51]

CRISPR-mediated dystrophin rescue in the dKO mouse after intravenous injection

The utility of systemic (intravenous [IV]) administration of the CRISPR AAVs was assessed in the dKO mouse model to determine whether broader targeting of muscle tissues could be achieved, and because this is a more widely used method for drug therapy delivery in patients. Several other studies have employed this route of administration for gene editing therapy in neonatal mice, demonstrating the utility of early intervention CRISPR AAV treatment.[23,24,27] An equivalent dose to that used for IP administration of CRISPR AAVs was delivered to P2 dKO mice via the facial vein (Figure 4B). Successful excision of *Dmd* exon 23 was detected by PCR in ∼81% (i.e., 13 out of 16) of the tissues analyzed (Figure 4A), and successful gene editing was confirmed by Sanger sequencing of the bulk edited PCR product (Figure 4B). Cloning of the edited amplicon and sequencing of five individual clones revealed both seamless editing and the formation of various random indels (Figure 4B), as is typical of repair by NHEJ.

The proportion of ΔExon23 *Dmd* mRNA transcripts was highest in the heart tissue at ∼5%, following IV administration of CRISPR AAVs (Figure 4C). Transcripts lacking *Dmd* exon 23 in the diaphragm, TA, and triceps were detected at a much lower abundance of ∼1.5% WT levels. Dystrophin protein, as measured by western blot, was partially restored in all four tissues (Figures 4D and 4E), with the highest expression in the heart and TA muscles (∼2% of WT levels) and lowest in the diaphragm and triceps (<1% of WT levels).

IF staining for dystrophin and laminin in transverse sections confirmed that dystrophin was partially restored in the heart, diaphragm, TA, and triceps muscles of neonatal dKO mice treated systemically with CRISPR AAVs by IV injection (Figure 5). Dystrophin-re-expressing myofibers were interspersed throughout the muscle in the heart, and to a lesser extent also the diaphragm, while in the TA and triceps there was distinct between-fiber patchiness (i.e., the sections consisted of both dystrophin-positive and dystrophin-negative myofibers). Within-fiber patchiness was also observed, whereby some dystrophin-positive myofibers exhibited incomplete dystrophin staining around the fiber circumference

of AAV-SaCas9 and 5 × 10^{11} vg of AAV-sgRNA and sacrificed at the humane endpoint. DNA, RNA, and protein were extracted from the heart (HRT), diaphragm (DIA), tibialis anterior (TA), and triceps (TRI) muscles. (C) Visualization of successful CRISPR-mediated *Dmd* exon 23 excision (ΔExon23 *Dmd*) in the gDNA by PCR and agarose gel electrophoresis. Samples exhibiting the desired editing outcome are highlighted with asterisks and corresponding indel frequencies indicated. (D) Edited PCR products were analyzed by Sanger sequencing, which confirmed successful exon 23 excision and fusion of the up- and downstream intronic regions following CRISPR AAV treatment. A representative sequencing chromatogram is shown for the bulk amplicon. Sequences illustrating specific indels are shown for individual cloned amplicons below the trace. PAM sites are labeled with yellow boxes. (E) RT-qPCR indicating the levels of ΔExon23 *Dmd* transcripts relative to WT. Western blots (F) and quantification of dystrophin protein restoration (G) relative to the vinculin protein loading control. Values are mean + SEM.

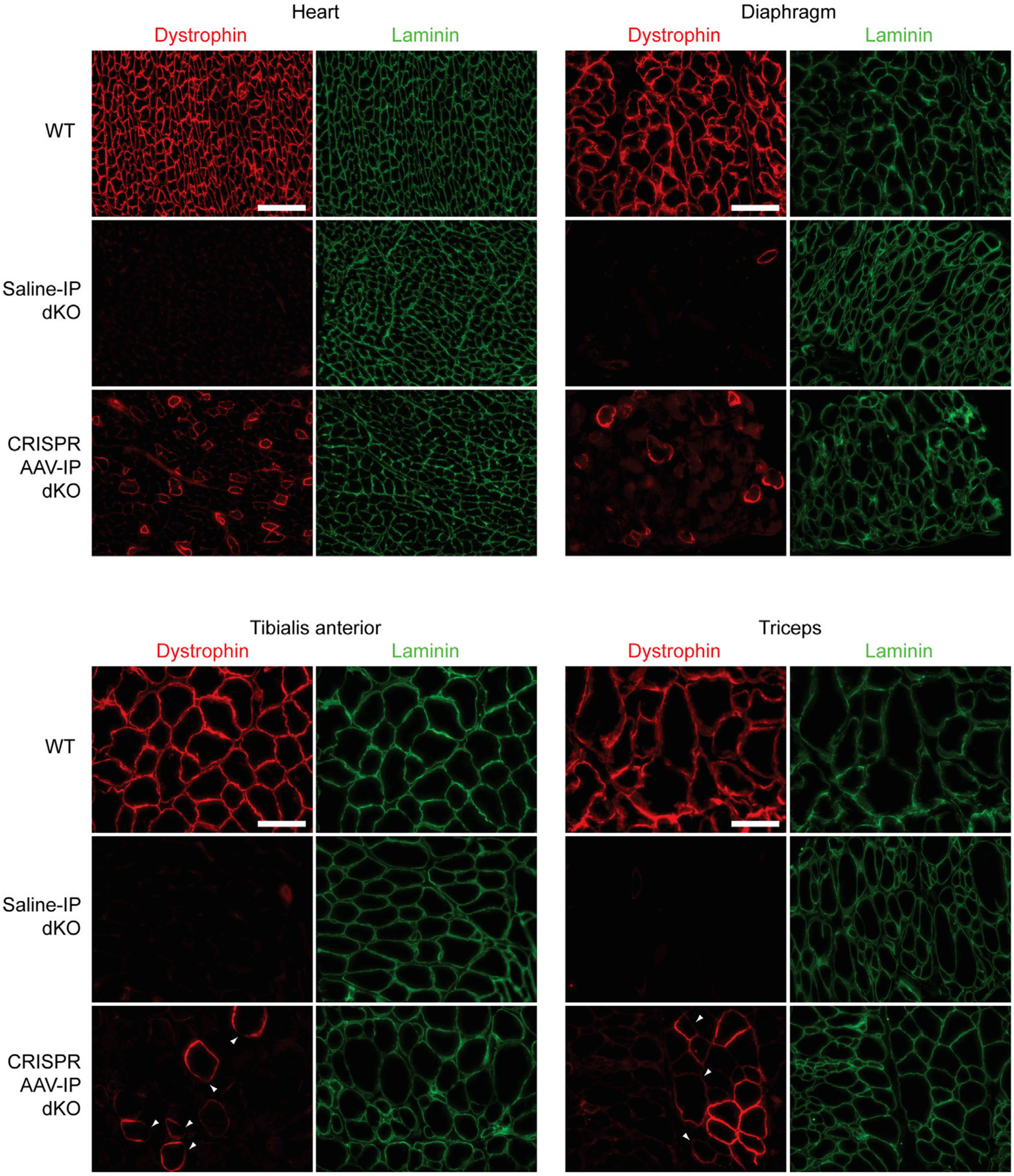

Figure 3. Dystrophin restoration in tissue sections from IP CRISPR AAV-treated dKO muscle

Representative dystrophin IF staining of transverse tissue sections obtained from heart, diaphragm, TA, and triceps muscles of dKO mice treated with CRISPR AAV9 particles by IP injection at P2. Untreated WT and saline-treated dKO samples were analyzed in parallel as controls. Sections were co-stained for laminin to delineate myofiber boundaries. Images were taken at 20× magnification. Scale bars represent 100 μm. Arrowheads indicate regions of patchy sarcolemmal dystrophin staining.

(where laminin co-staining was clearly present) (Figure 5, arrowheads).

Dystrophin gene editing did not improve dKO lifespan

No difference in the weight gain of the CRISPR AAV-treated dKO mice was observed relative to controls (Figure 6A). Furthermore, the average lifespan of the AAV-IP and -IV treatment groups was ~7-weeks and ~8-weeks, respectively (Figure 6B). This was not significantly different to that of the control dKO mice which also succumbed to the dystrophic pathology around the age of 8 weeks ($p = 0.7857$ and >0.9999 for the AAV-IP and AAV-IV treatment groups relative to the saline control, respectively). Similarly, serum miRNA biomarkers were not restored to WT levels in the CRISPR-treated animals (Figure S2).

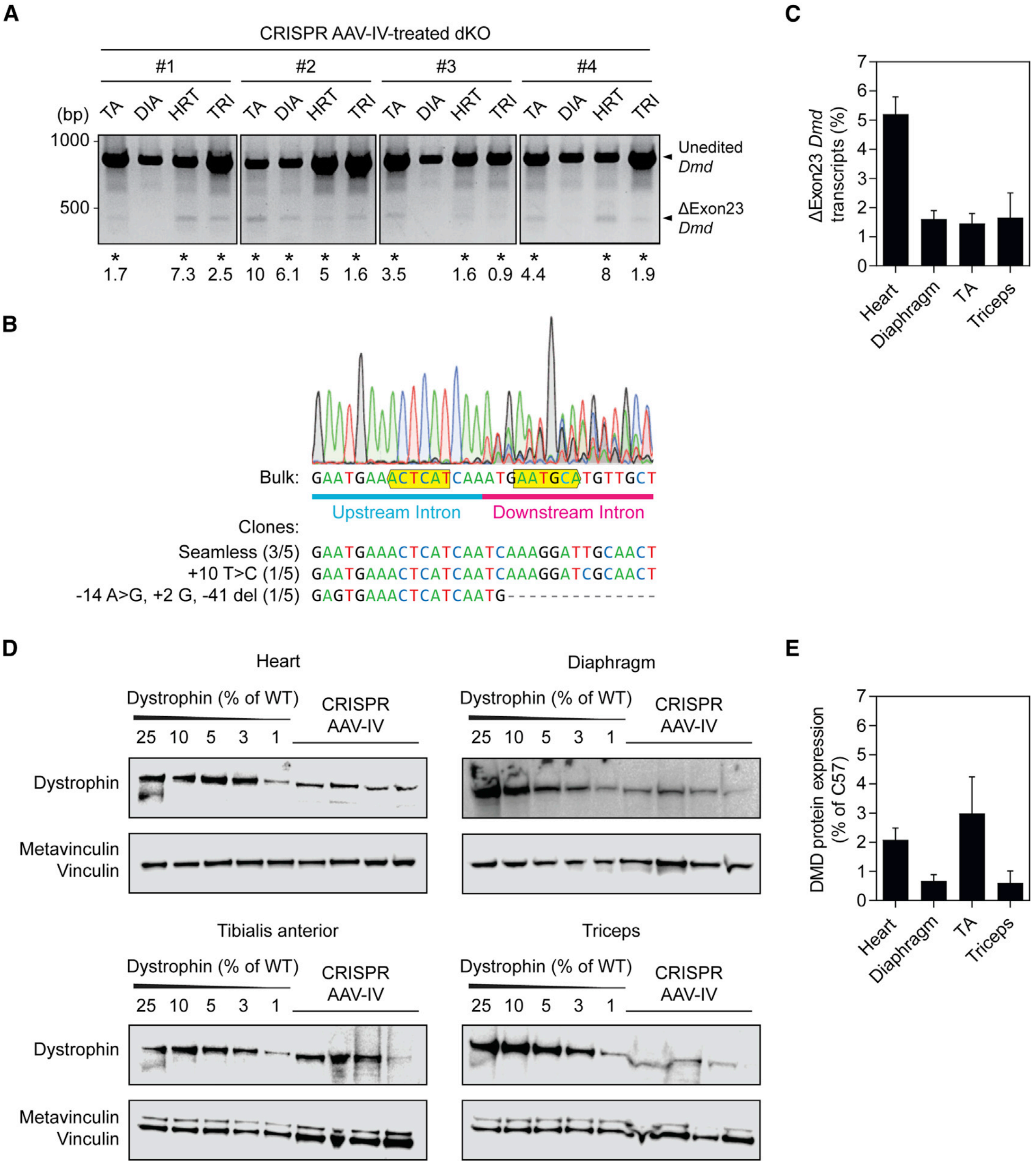

Figure 4. Evaluation of *Dmd* exon 23 excision and dystrophin restoration in dKO mice after IV injection of neonates

dKO mice were treated by IV injection (n = 4) via the facial vein at postnatal day 2 (P2) with 1×10^{11} vector genomes (vg) of SaCas9-AAV and 5×10^{11} vg of dual sgRNA-AAV and sacrificed at the humane endpoint. DNA, RNA, and protein were extracted from the heart (HRT), diaphragm (DIA), TA, and triceps (TRI) muscles. (A) Visualization of successful CRISPR-mediated *Dmd* exon 23 excision (ΔExon23 *Dmd*) in the gDNA by PCR and agarose gel electrophoresis. Samples exhibiting the desired editing outcome are highlighted with asterisks and corresponding indel frequencies indicated. (B) Edited PCR products were analyzed by Sanger sequencing, which confirmed successful exon 23 excision and fusion of the up- and downstream intronic regions following CRISPR AAV treatment. A representative sequencing chromatogram is shown for the bulk amplicon. Sequences illustrating specific indels are shown for individual cloned amplicons below the trace. PAM sites are labeled with yellow boxes. (C) RT-qPCR indicating the levels of ΔExon23 *Dmd* transcripts relative to WT. Western blots (D) and quantification of dystrophin protein restoration (E) relative to the vinculin protein loading control. Values are mean + SEM.

The biodistribution of AAV vectors delivered by either IP or IV delivery routes was assessed by measuring the number of vector genome copies (using primers against the SaCas9 transgene) per host genome by absolute quantification qPCR (Figure S3). AAV vectors were present at the highest levels in the heart and diaphragm tissues for both IP and IV injected animals, consistent with gene editing

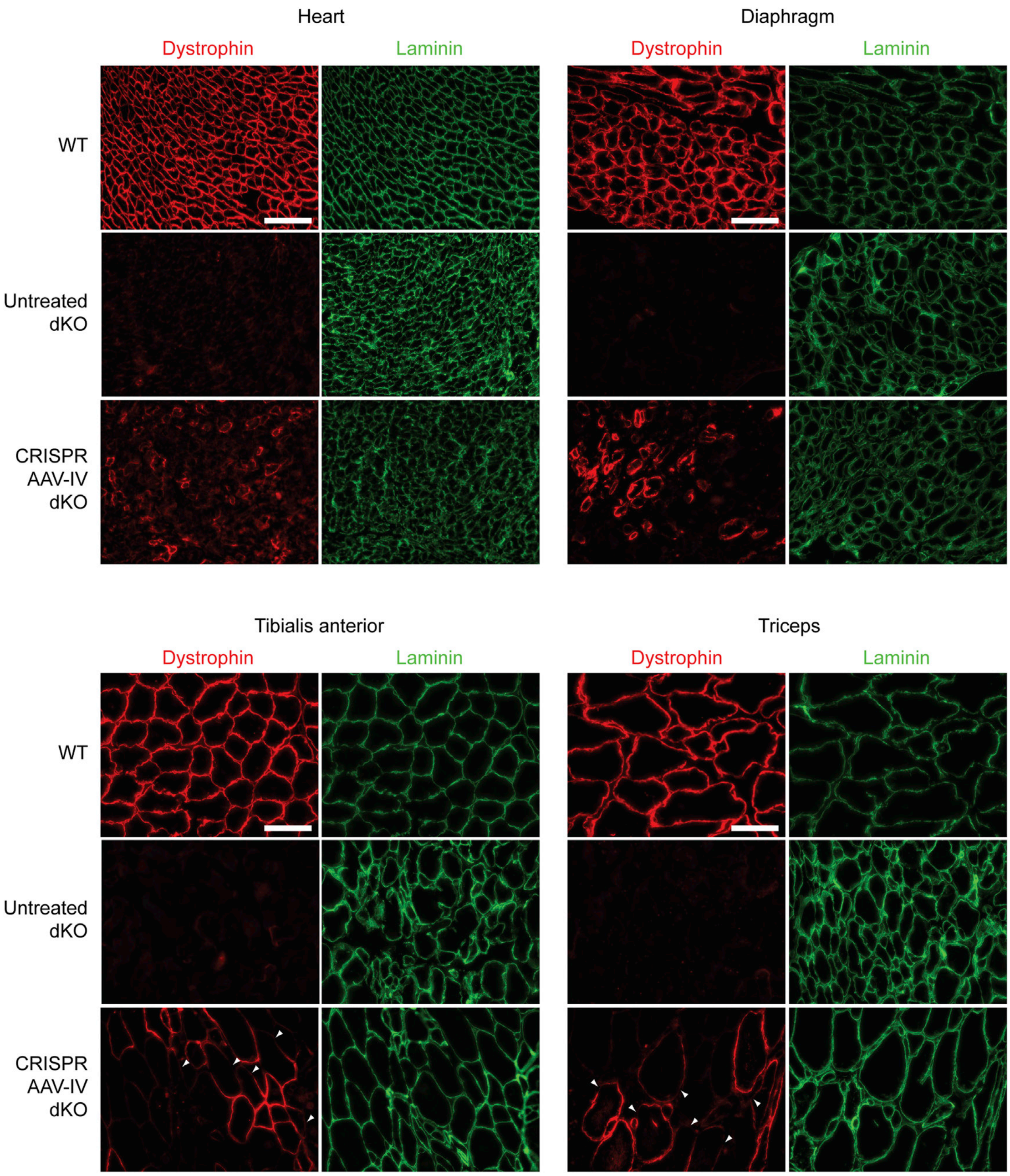

Figure 5. Dystrophin restoration in tissue sections from IV CRISPR AAV-treated dKO muscle

Representative dystrophin IF images of transverse tissue sections obtained from heart, diaphragm, tibialis anterior, and triceps muscles of dKO mice treated with CRISPR AAV9 particles by IV injection at P2. Untreated WT and saline-treated dKO samples were analyzed in parallel as controls. Sections were co-stained for laminin to delineate myofiber boundaries. Images were taken at 20× magnification. Scale bars represent 100 μm. Arrowheads indicate regions of patchy sarcolemmal dystrophin staining.

outcomes. Notably, the levels of AAV vector genomes in the TA and triceps muscles were very low in the IP-treated mice, and between 26- and 95-fold lower than in the same tissues from IV-treated mice (Figure S3). These data suggest that the IV route of administration is important for enabling delivery to peripheral muscles.

Non-uniform dystrophin localization in dystrophic muscle after CRISPR AAV treatment

We have previously reported on the importance of uniform dystrophin expression for correcting dystrophic pathology and preventing the release of circulating miRNA biomarkers using the *mdx-Xist*$^{\Delta hs}$

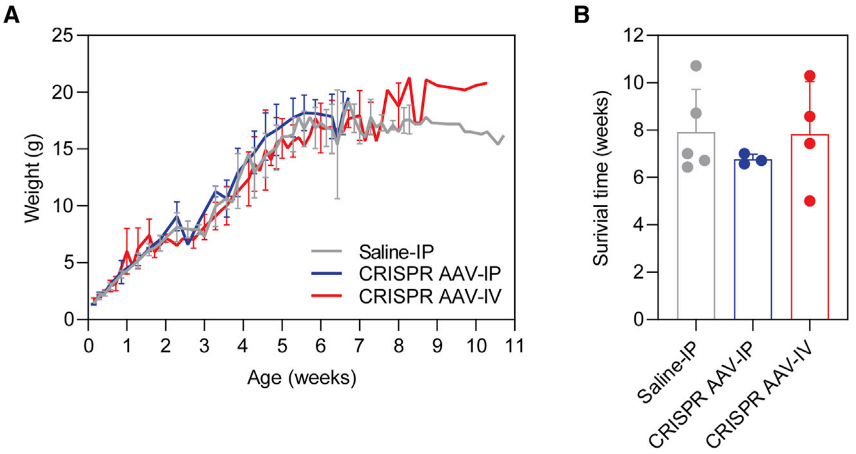

Figure 6. CRISPR AAV treatment does not improve lifespan in dKO mice

dKO mice were treated with saline (n = 5) or CRISPR AAV vectors by IP (n = 3) or IV (n = 4) injection routes and weighed three times weekly until culling at the humane endpoint. (A) Animal body weight measurements over time; values are mean ± SEM. (B) Age at humane endpoint (i.e., survival time); values are mean + SEM.

mouse (which expresses dystrophin in a patchy manner as a consequence of skewed X chromosome inactivation).[52] In the present study, some degree of within-fiber patchiness was observed in transverse sections (Figures 3 and 5, arrowheads), although this phenomenon is typically more easily seen in longitudinal sections. We therefore next sought to analyze the pattern of dystrophin expression in longitudinal sections of TA muscles derived from dKO mice that received CRISPR AAV treatment by either IP and IV route of injection (Figure 7). Such a patchy dystrophin distribution was observed for both treatment regimens, whereby adjacent regions of dystrophin-positive and dystrophin-negative sarcolemma were observed within the same myofiber (Figure 7).

Given the importance of dystrophin in acting as an organizing center for the dystrophin-associated protein complex (DAPC),[53] we were motivated to assess the pattern of sarcolemmal distribution of various DAPC proteins in CRISPR-treated dKO mice by IF. β-dystroglycan (DAG1) was expressed at low levels throughout dKO TA muscle fibers after injection with the CRISPR AAV vectors. However, regions of intense staining were observed that colocalized with dystrophin-positive sections of sarcolemma for both transverse and longitudinal orientations (Figure S4). These findings are similar to those reported by Scaglioni et al., who investigated dystrophin and β-dystroglycan expression in muscle biopsy samples from DMD patients treated with golodirsen (an exon-skipping drug designed to skip *DMD* exon 53).[54] In this study, β-dystroglycan was expressed at low levels throughout the biopsy sections, and staining was positively correlated with dystrophin expression. The additional DAPC factors, DTNA (α-dystrobrevin) (Figure S5) and NOS1 (neuronal nitric oxide synthase [nNOS]) (Figure S6) also exhibited patchy patterns of expression as visualized in both transverse and longitudinal orientations. Notably, we have previously observed within-fiber patchy patterns of protein distribution for DTNA and NOS1 in *mdx-Xist*$^{\Delta hs}$ muscle tissues.[52] These data suggest that non-uniform, spatially-restricted dystrophin re-expression is associated with similar non-uniform, within-fiber patchy recovery of DAPC expression.

Detection of on-target, non-productive editing events by long-read sequencing

The patchy pattern of sarcolemmal dystrophin distribution is likely a consequence of the chimeric nature of the treated fibers, which contain both corrected (i.e., dystrophin-expressing) and uncorrected (i.e., non-dystrophin-expressing) myonuclei. This scenario may arise as a result of incomplete gene editing, whereby the CRISPR-Cas9 machinery does not reach all myonuclei within a fiber. However, it is also expected that not all gene editing events will be repaired in a productive manner, meaning that the *Dmd* locus may be incorrectly edited in some myonuclei, such that they can never express functional dystrophin protein. To characterize the degree of non-productive gene editing, we performed targeted long-read sequencing of an ∼800 bp PCR amplicon covering *Dmd* exon 23 together with flanking intron sequence containing both sgRNA target sites using the PacBio sequencing platform. This analysis was performed in cardiac muscle, as gene editing was most effective in this tissue, and so these samples were expected to contain the most complex sequencing libraries. Sequencing reads were processed using a custom pipeline in order to assign them as unedited, productive edited (i.e., excision of *Dmd* exon23), and non-productive edited. Amplicons exhibiting non-productive editing were further classified as having (1) disruption of the 5' sgRNA target site, (2) disruption of the 3' sgRNA target site, (3) disruption of both sgRNA target sites, (4) inversion of the excised fragment, and (5) AAV vector backbone integration (Figure 8A). The success of the classification was assessed by determining the amplicon size distributions for the classification bins (Figure S7).

The majority (82%–86%) of reads in the CRISPR-treated libraries were unedited, whereas productive excision of *Dmd* exon 23 was observed at a rate of 0.43%–0.67% based on this analysis (Figure 8A). In contrast, only 0.03% of reads in the control sample were categorized as lacking *Dmd* exon 23. (Note: this analysis is expected to underestimate the degree of productive editing as the PCR reaction was optimized to preferentially amplify the full-length target.).

A major limitation of the dual sgRNA targeting exon excision CRISPR strategy used in this study is that the success of this approach is predicated on simultaneous cleavage at both target sites. However, asymmetric cleavage, whereby one site is cut and repaired forming an indel before the neighboring site can be cut, will result in a non-productive gene editing outcome and render the locus refractory to

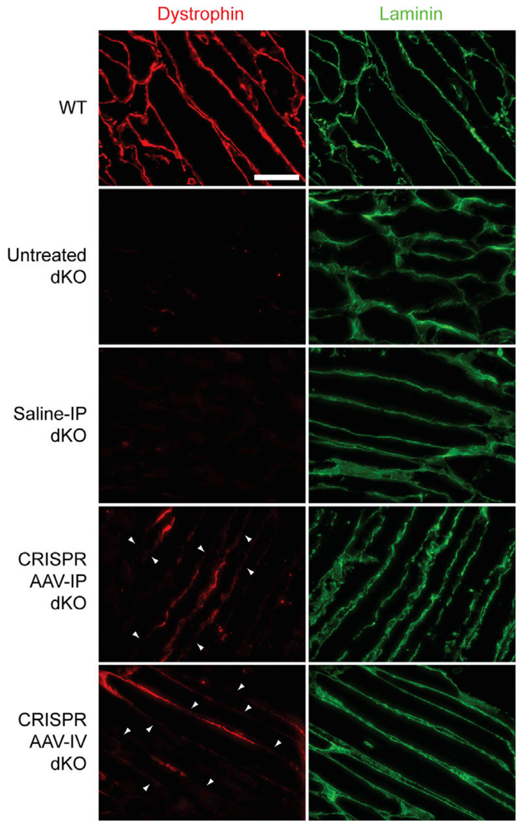

Figure 7. Dystrophin protein is re-expressed in a within-fiber patchy manner in dKO muscle after CRISPR AAV treatment

Representative dystrophin IF staining in longitudinal TA muscle sections of dKO mice treated with CRISPR AAV9 particles by IP or IV injection at P2. Untreated WT, untreated dKO, and saline-treated dKO samples were analyzed in parallel as controls. (The pictured CRISPR-treated sections are derived from animals that express 1% and 3.3% of WT dystrophin for IP- and IV-treated animals respectively.) Sections were co-stained for laminin to delineate myofiber boundaries. Images were taken at 20× magnification, and scale bars represent 100 μm. Arrowheads indicate regions of patchy sarcolemmal dystrophin staining.

further editing. Asynchronous indel formation was observed at both the 5' and 3' sgRNA target sites, although the levels of such non-productive editing were much higher for the 5' site (9.9%–13.4%) (Figure 8A). While indels were detected at the 3' sgRNA site, these were present at levels similar to the saline-treated control sample (i.e., background) (~2%) (Figure 8A). These data suggest that CRISPR-mediated cleavage occurred much more efficiently at the 5' sgRNA cut site than for the 3' site. Similar results were obtained when these sites were analyzed by Sanger sequencing deconvolution using the inference of CRISPR edits (ICE) method (Figures 8B and 8C). We also detected reads in which indels were formed at both sgRNA sites, indicative of cleavage and repair, although the intervening *Dmd* exon 23 sequence was not excised. The relatively high percentage of asynchronous, non-productive indel formation at the 5' sgRNA target site is likely a major factor limiting the efficacy of this gene editing therapeutic approach.

A very small proportion of reads (~0.03%–0.06%) contained inversions of exon 23, whereby the region between the two cut sites was excised and then re-inserted in the reverse orientation (Figure 8A).

The CRISPR-treated libraries also contained a substantial number (0.38%–1.11%) of reads containing AAV-derived sequences (Figure 8A). These reads consisted of amplicons containing AAV vector backbone sequence (obsvered for both vectors), and in both forward or reverse orientations. Notably, this rate of AAV integration events is likely to be an underestimate, as the PCR reaction is expected to be biased against the amplification of the larger template sequence resulting from full-length AAV backbone integration. Consistent with this notion, reads in the AAV integration classification bin exhibited shift toward a larger amplicon size, which was not observed in other read categories, but was still much shorter than the full-length SaCas9-AAV genome (maximum detected size, 1,721 bp; Figure S7). The presence of AAV integration events was further confirmed by PCR of gDNA using a forward primer located in intron 22 and a reverse primer located in the CMV promoter of the AAV-SaCas9 vector, or in the U6 promoter of the AAV dual sgRNA vector, as appropriate (Figure 8D). PCR amplicons of the expected size were only detected for CRISPR-treated animals, and Sanger sequencing of clones of these amplicons revealed the presence of AAV genome sequence as expected, although a variety of fragmentations were observed (Figure S8). Amplicon reads that were assigned to the *Dmd* exon 23 inversion or AAV integration event categories were extremely low in the saline-treated sample (i.e., 0% and 0.0022%, respectively). Taken together, these data are indicative of frequent, non-productive editing events in CRISPR-treated cardiac muscle.

DISCUSSION

Here we demonstrate CRISPR-mediated rescue of dystrophin expression in the severely affected dKO mouse model of DMD. Several other studies have employed a similar dual sgRNA CRISPR exon excision strategy for dystrophin restoration,[23–27,31,55] although these have typically utilized the *mdx* mouse model, which exhibits only a mild phenotype and almost normal lifespan.[23–25,27] While we observed dystrophin restoration in multiple dKO muscle tissues (Figures 2, 3, 4, 5, and 7), this was not sufficient to improve survival (Figure 6). In contrast, exon skipping using antisense oligonucleotides[48,56] and the U7 snRNA system[49] has been shown to result in pronounced increases in lifespan from ~8 to 10 weeks in untreated animals to between >6 months[48,56] and ~1 year,[49] respectively (although the levels of dystrophin expression reported in these reports were much higher than that observed in the present study).

We observed up to 5% and 5.7% of WT dystrophin protein levels in the heart and diaphragm muscles of dKO mice that received CRISPR AAVs by IP injection, and up to 2.9% and 6.1% in the heart and TA muscles of those treated by IV administration, respectively (Figures 2 and 4). Comparable levels of dystrophin have previously been reported in the hearts of CRISPR-treated *mdx* mice assessed at 3 weeks[24] and 8 weeks[23,25,27] post treatment (i.e., a similar time frame to the limited lifespan of the dKO mouse). Notably, several *mdx* studies

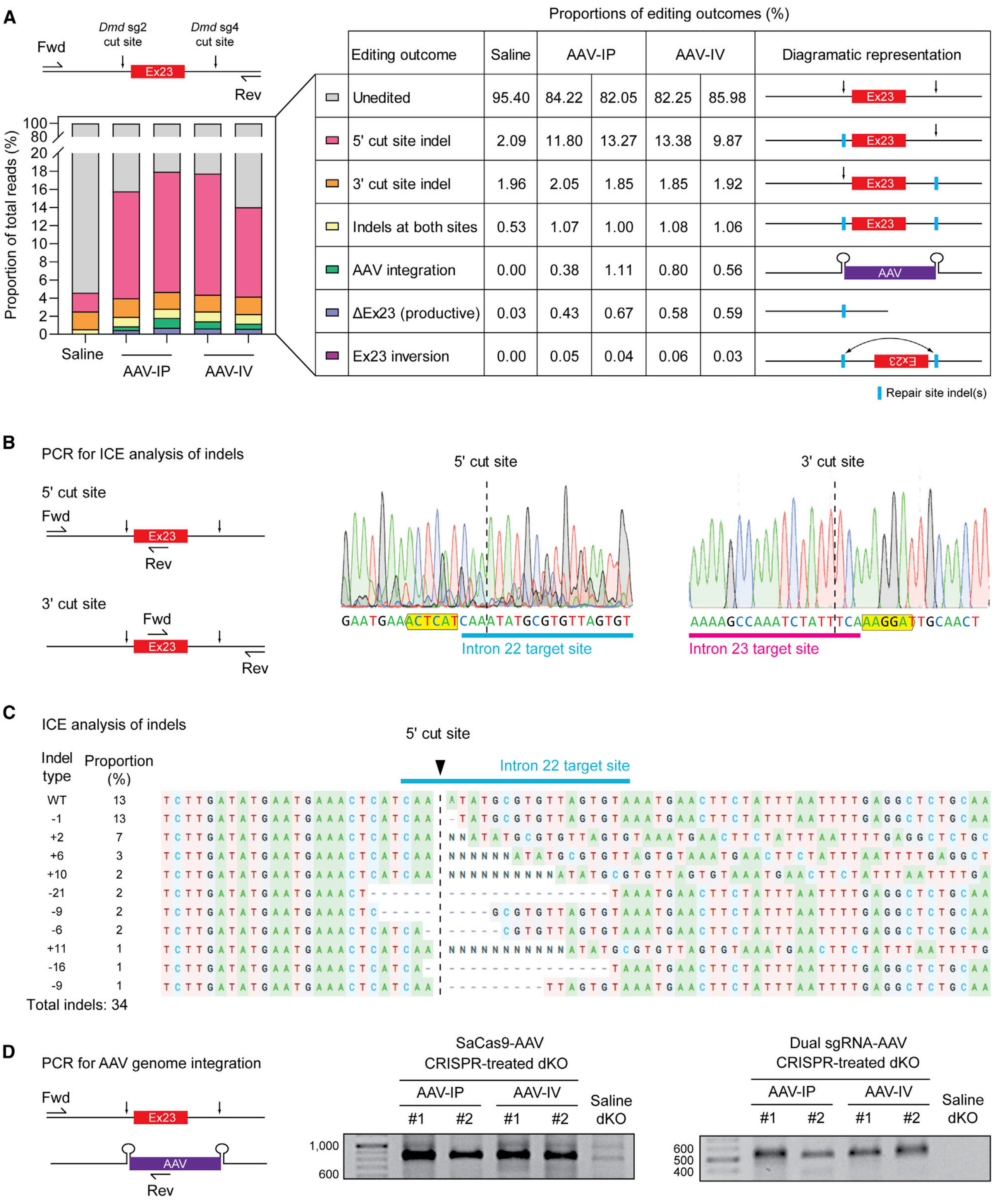

Figure 8. Detection of productive and non-productive editing outcomes by long-read DNA sequencing

DNA was harvested from dKO mice following treatment with CRISPR AAV (administered via either IP or IV routes) and the edit region amplified by PCR (n = 2 each). Saline-injected dKO tissue was collected in parallel as a control (n = 1). Heart muscle was used in all cases as editing was found to be highest in this tissue. (A) PCR amplicons were

(legend continued on next page)

reported progressive increases in dystrophin protein levels at 6 months,[23] 12 months,[25] and 18 months[31] post treatment, with up to ∼20% restoration in the heart at the latest time point.[31] It has been suggested that this accumulation of dystrophin is a consequence of its long half-life (>100 days)[57,58] and of corrected myofibers/cardiomyocytes experiencing a selection advantage over dystrophin-negative, unprotected fibers that remain sensitive to contractile damage and myonecrosis.[59] In contrast, it is likely that the treated dKO animals used in the present study died before there had been sufficient time for corrected dystrophin protein to accumulate to levels above the threshold required for phenotypic correction. Indeed, the levels of dystrophin protein restoration that we observed fell short of those that have been reported to be required for phenotypic correction. Specifically, X-linked cardiomyopathy patients with only 29% of normal dystrophin levels exhibit sub-clinical myopathy.[60] Re-expression of dystrophin by either exon skipping[61] or expression of a minidystrophin[62] resulted in phenotypic correction at levels of 15% or 20%–30% of WT respectively in *mdx* mice. Notably, the levels of dystrophin observed after CRIPSR/Cas9 treatment are similar to those reported in human DMD patients treated with the FDA-approved exon-skipping drug eteplirsen (i.e., treated patients expressed 0.93% dystrophin relative to healthy controls).[18]

The dose of each AAV9 vector that we utilized (1×10^{11} of SaCas9-AAV, and 5×10^{11} vg of dual sgRNA-AAV, a dose that is equivalent to $\sim 5 \times 10^{13}$ and 2.5×10^{14} vg/kg respectively) is comparable with that used in published *mdx* studies at the time that this study was initiated.[23,24,27] However, it has since emerged that both the dose of the AAV as well as the ratio of sgRNA:Cas9 are important determinants of gene editing success. The effect of increasing the dose of vectors carrying the CRISPR machinery was demonstrated by Bengtsson and colleagues, where the authors assessed co-excision of "hotspot" exons 52 and 53 in the mdx^{4cv} mouse (which harbors a nonsense mutation within exon 53).[26] The authors observed that widespread skeletal muscle restoration was only observed at the highest dose (i.e., 1×10^{13} vg of SpCas9-AAV and 4×10^{12} of sgRNA-AAV).[26] It is therefore possible that injection of higher doses of our vectors may result in improved dystrophin restoration and functional correction in the dKO mouse.

The dose of AAV required to achieve therapeutic dystrophin editing in patients is currently unknown, but based on pre-clinical studies, is likely to be high. This raises the issue of whether sufficient quantities of virus can realistically be produced to treat all patients given current manufacturing demands/constraints. Furthermore, the feasibility of using high-dose AAV regimens in patients has been called into question as important safety concerns have recently emerged, specifically in light of the deaths of child participants in a clinical trial for X-linked myotubular myopathy (NCT03199469),[63] and another child participant in a separate AAV trial for mucopolysaccharidosis type IIIA (NCT03612869).[64] Furthermore, during the initial preparation of this manuscript, Pfizer announced that a DMD patient treated with high-dose AAV microdystrophin gene therapy had died, although few other details have been publicly disclosed at the time of writing.[65] Additionally, during the revision of this manuscript, Novartis reported the deaths of two patients receiving zolgensma (an AAV gene therapy for spinal muscular atrophy [SMA]) as a consequence of acute liver failure.[66]

The dose of AAV administered could be reduced if more potent strategies are used, such as self-complementary AAV (scAAV). scAAV vectors form dsDNA hairpin structures, and thereby bypass the rate-limiting second-strand synthesis step in the AAV life cycle, leading to accelerated transgene expression.[67,68] Zhang and colleagues observed a 70-fold improvement in editing efficiency in *mdx* mice with sgRNAs expressed from scAAV relative to conventional AAV at the same dose when applying a 1:10 ratio of AAV-Cas9 to AAV-sgRNA.[33] However, we also performed additional injections using our vectors at this 1:10 ratio but observed negligible dystrophin restoration and no lifespan extension (data not shown). Alternative approaches to enhancing gene editing potency include improved capsids with stronger muscle tropism, de-targeting of vectors from the liver, higher Cas9/sgRNA expression, and better sgRNA design.

Assuming that CRISPR therapies can be safely developed for the treatment of DMD patients, the high doses of AAV required will undoubtedly be associated with a substantial price tag. For comparison, zolgensma (a single-vector treatment) is the most expensive single-dose drug at US$2.1 million approved for use in SMA patients less than 2 years old. CRISPR therapies for DMD may require multiple vectors, and will most likely be applied to patients older than 2 years, which further inflates costs. Given these safety issues, and the high cost associated with multiple vector approaches, classical gene therapy (e.g., with microdystrophin) may be preferable to CRISPR-Cas9 approaches as a single vector can theoretically be used to treat all patients. Notably, the high price of AAV-based therapies may be justified if improvements in patient health are sufficient to result in a reduction in the cost of care in the years following treatment.

Analysis of longitudinal sections of CRISPR-treated dKO mouse skeletal muscle tissue revealed a within-fiber patchy pattern of dystrophin restoration following CRISPR treatment (Figure 7), a phenomenon that we had previously predicted.[34,52,69] The observed patchiness is consistent with the myonuclear domain hypothesis, whereby each myonucleus serves its proximal cytoplasmic territory.[70] To our knowledge, no other DMD CRISPR study has analyzed dystrophin

analyzed by long-read next-generation DNA sequencing and assigned to one of the following categories: unedited, indels at either sgRNA target site (or both), presence of integrated AAV backbone, productive excision of *Dmd* exon 23 (ΔEx23), and inversion of exon 23. The percentages of each editing outcome are indicated in the table. (B) The formation of indels was assessed at both 5' and 3' cut sites by PCR and Sanger sequencing. (C) The 5' cut site was further analyzed using the inference of CRISPR edits (ICE) to characterize indel formation. (D) Integration of AAV backbones derived from both SaCas9-AAV and dual sgRNA-AAV vectors was confirmed in the treated animals by PCR and Sanger sequencing of the 5' cut site (Figure S8).

immunostaining in longitudinal muscle sections. The functionality of muscle relies on coordinated contraction and force dissipation along the length of the myofiber, and, as such, incomplete sarcolemmal dystrophin coverage following gene editing could result in a failure to achieve functional correction. The role of dystrophin is to protect myofibers from contractile damage,[71] so, in the case of patchy patterns of sarcolemmal dystrophin expression, molecular pathological events could still initiate at unprotected dystrophin-negative regions within the myofiber. These observations may partially explain why no lifespan extension was observed in this study. Interestingly, Torelli et al. recently reported incomplete sarcolemmal dystrophin coverage in human patients with severe BMD and intermediate muscular dystrophy (i.e., disease severity greater than typical BMD, but less severe than typical DMD)[68] which provides support for the notion that dystrophin uniformity is likely to be a factor in the success of therapeutic interventions in patients.

A similar patchy dystrophin pattern was observed in the *mdx-Xist*$^{\Delta hs}$ mouse, which expresses varying levels of dystrophin as a result of skewed X chromosome inactivation.[52,72] The myofibers of both the *mdx-Xist*$^{\Delta hs}$ mouse and CRISPR-treated dKO mice are heterokaryons that contain both dystrophin-expressing, and non-dystrophin-expressing myonuclei, which likely explains the similarity between these model systems. These findings strongly suggest that dystrophin mRNA and protein are subject to a degree of spatial restriction and are not free to diffuse throughout the sarcoplasm and sarcolemma, respectively. While the levels of dystrophin restoration in our CRISPR-treated animals was low, patchiness in the *mdx-Xist*$^{\Delta hs}$ model was observed even with >40% of WT dystrophin expression levels.[52] We therefore propose that the *mdx-Xist*$^{\Delta hs}$ mouse is a putative model that simulates the outcome of CRISPR-mediated dystrophin correction over a range of effectiveness levels. In contrast, we have observed that antisense oligonucleotide-mediated exon skipping with peptide-phosphorodiamidate morpholino oligonucleotide (PMO) conjugates results in within-fiber uniform dystrophin distribution that is independent of dose (see related manuscript).[73]

Notably, van Putten and colleagues generated a variant dKO model crossed with the *Xist*$^{\Delta hs}$ mouse (i.e., *mdx*/*utrn*$^{-/-}$/*Xist*$^{\Delta hs}$).[74] In this model, an increase in survival was observed in mice expressing as little as ~4% WT dystrophin levels.[74] Crucially, these mice experienced the restorative benefits of body-wide expression of full-length dystrophin protein from birth, whereas CRISPR treatment in the present study resulted in the generation of a BMD-like dystrophin, with a partial internal truncation and attenuated functionality,[75] which was differentially restored across muscle tissues. Additionally, the *mdx*/*utrn*$^{-/-}$/*Xist*$^{\Delta hs}$ mice are necessarily female (as variable dystrophin expression is dependent on skewed X chromosome inactivation), which is a further potentially confounding factor. Importantly, this study demonstrates that the failure of our CRISPR treatments to extend lifespan is likely a consequence of inadequate effectiveness of the therapy to achieve sufficient dystrophin expression, and not an intrinsic utrophin-related issue (e.g., neuromuscular junction dysfunction). Nevertheless, while the dKO mouse is useful given the severity of its pathology, we acknowledge the inherent limitation of using a model that carries a "genetic insult" that is distinct from that observed in DMD patients.

Instances in which Cas9-mediated cleavage occurs and the DSB is repaired in a non-productive manner (i.e., corruption of one or more of the sgRNA target sites,[55] inversion of excised region,[76] or integration of sequence from the AAV vector genome)[25,32,77] are likely to contribute to myofiber heterogeneity as the resulting myonuclei are incapable of producing dystrophin or being re-cut by Cas9. Such non-productive editing events were readily detected in the heart tissue of CRISPR-treated dKO animals (Figure 8). In particular, we observed a relatively high proportion of indel formation at the 5' sgRNA cut site, indicative of asynchronous cleavage and repair. Such nuclei are rendered refractory to further editing as the introduction of an indel disrupts the sequence of the sgRNA binding site. This observation of differential sgRNA cutting efficiency was somewhat surprising, given that initial *in vitro* screening suggested that both guides were effective (Figure 1). These data are also suggestive of differential cleavage potential for the 5' and 3' sgRNAs, which is likely limiting the overall dystrophin restoration efficacy of this therapeutic approach.

Importantly, while there is a possibility of CRISPR-induced lesions being resolved in a non-productive manner, the generation of myofiber heterokaryons is inevitable. In other words, even if DNA cleavage is achieved in every single myonucleus, some of these will be non-productively repaired, meaning that the treated fibers must necessarily consist of both dystrophin-expressing and non-dystrophin-expressing nuclei. An alternative to entire exon excision is the use of a single-cut CRISPR strategy to disrupt splice acceptor sites and putative exonic splicing enhancer motifs in the gDNA. As a result, the spliceosome is no longer able to recognize these splicing signals, thereby achieving permanent exon skipping. This strategy has been utilized in *mdx* mice,[26,29,32,78] as well as a canine model of dystrophy,[30] with high levels of gene editing success. For this approach, the formation of an indel at the cut site is expected to result in restoration of dystrophin expression, whereas seamless repair without an indel regenerates the sgRNA target site. As such, the single-cut CRISPR strategy may be superior to the dual-cut approach owing to the simplicity of design and propensity for a greater number of productive editing outcomes. However, the single-cut strategy cannot be leveraged for multi-exon excision. Furthermore, AAV genome integration is still possible at the DSB site for the single-cut approach, which is likely to result in a non-productive editing outcome. Similarly, the integration of gene regulatory sequence from the AAV genome in the *Dmd* gene body may result in ectopic transcription initiation and/or termination events that disrupt dystrophin expression.

While terminally differentiated myofibers are the primary target of DMD gene editing therapies, editing of the muscle satellite (stem) cell pool would be highly beneficial. Permanent correction of the dystrophin locus in these muscle resident cells is highly desirable, as these cells would continue to add dystrophin-expressing myonuclei to the

mature fibers during growth and regeneration throughout the life of the treated individual. As a result, an enrichment of dystrophin expression would be expected over time. It has been reported that AAV vectors are incapable of transducing satellite cells.[79] However, Tabebordbar et al. reported AAV-mediated CRISPR correction in satellite cells, although at relatively low efficiencies.[24] Given the low levels of gene editing observed in this study, we did not investigate editing in satellite cells (where levels would be expected to be below the limit of detection).

CRISPR-Cas9-based therapies have the potential to activate the adaptive immune system through the presentation of therapy-associated antigens (i.e., Cas9 and the re-expressed truncated dystrophin proteins) to T cells and/or the production of neutralizing antibodies. Such anti-transgene responses are likely to diminish the effectiveness of the therapy but can be avoided by initiating treatment as early as possible. To this end, Nelson et al., showed that anti-SaCas9 antibodies were present 6 months post treatment in the serum of adult *mdx* mice, but were absent at 1 year post treatment in *mdx* mice treated at the neonatal stage.[25] Similarly, vector genome copies declined in the adult-treated animals but persisted in the neonatal-treated animals.[25] Although laboratory animals can be treated at P1 or P2 (as in the present study) with ease, initiating therapies this early in human patients constitutes a substantial challenge. Similar anti-Cas9 immune responses have also been reported in canine DMD models.[80] As such, anti-transgene immune responses may still present a significant barrier to effective therapy development. Importantly, there is evidence that pre-existing anti-Cas9 antibodies and Cas9-reactive T cells are relatively common in the general population,[81,82] which constitutes a further obstacle for the effective translation of CRISPR therapies in human patients.

In conclusion, CRISPR-mediated dystrophin restoration in the severely affected dKO mouse did not reach a sufficient level to achieve an improvement in survival. Future developments are required to increase the overall editing efficiency of the CRISPR AAV dual-cut strategy employed in this study, including optimization of delivery to a broad range of muscle tissues, particularly the heart and diaphragm, and reducing the likelihood of non-productive editing outcomes. This study highlights the importance of uniform dystrophin expression in the successful implementation of genetic therapies for DMD.

MATERIALS AND METHODS

Plasmids and AAV production

For cell culture studies, guide RNAs were expressed using pTZ-U6sgRNA(SaCas9) vectors that were modified to encode specific *Dmd*-targeting sequences (Table S1) by Golden Gate Assembly.[83] SaCas9 was expressed using pX601-AAV-CMV::NLS-SaCas9-NLS-3xHA-bGHpA; U6::BsaI-sgRNA), which was a gift from Feng Zhang (Addgene plasmid #61591; http://n2t.net/addgene:61591; RRID: Addgene_61591).[41] AAV2/9 vectors were generated by Atlantic Gene Therapies (University of Nantes, Institut de Recherche Thérapeutique, Nantes, France) using pX600 (SaCas9-AAV) (Addgene, Watertown, MA, USA) and pAAVio-2x.sgRNA (*Dmd* dual sgRNA-AAV).

Cell culture

10T1/2 mouse fibroblasts cells (a kind gift from Peter K. Vogt) were cultured in DMEM supplemented with 10% FBS and 1× antibiotic/antimycotic (all Thermo Fisher Scientific, Abingdon, UK) and maintained in a humidified incubator at 37°C with 5% CO_2. Cells were seeded at 1×10^5 cells per well in a 24-well multiplate and transfected with 800 ng of plasmid DNA per well (400 ng of SaCas9 expression plasmid and 200 ng of each sgRNA plasmid) using Lipofectamine 2000 (Thermo Fisher Scientific) according to manufacturer's instructions, and harvested 48 h later.

Animal studies

All procedures were authorized by the UK Home Office (project license 30/2907) in accordance with the Animals (Scientific Procedures) Act 1986. Animals were housed under 12-h light/12-h dark conditions, with access to food and water *ad libitum*.

Utrophin/dystrophin dKO mice were obtained in the F1 generation by breeding with *Utrn*^{tm1Ked}*Dmd*^{mdx}/J mice (JAX stock #019014; Jackson Laboratory, Bar Harbor, ME, USA) which are heterozygous for the utrophin-knockout mutation and homozygous (females)/hemizygous (males) for the *mdx* dystrophin-knockout mutation.[46] WT C57BL/10 (C57BL/10ScSn) and dystrophic *mdx* (C57BL/10ScSn-*Dmd*^{mdx}/J) mice were also used as controls as appropriate.

dKO mice were treated at P2, using either the IP (n = 3) or IV (via the facial vein, n = 4) injection routes, with 20 μL of a mixture of the two AAV vectors in 0.9% sterile saline. The vectors were formulated as a 1:5 SaCas9:sgRNA mixture consisting of 1×10^{11} vg SaCas9-AAV and 5×10^{11} vg *Dmd* dual sgRNA-AAV. Control dKO mice were treated with 0.9% sterile saline (n = 5).

Mice were weighed three times a week, and sacrificed at the humane endpoint (which was determined by rapid labored breathing and reduced mobility leading to difficulty accessing food and water) by increasing CO_2 concentration. Serum was collected from the jugular vein immediately after termination at the humane endpoint, using Microvette CB 300 Blood Collection tubes (Sarstedt, Nümbrecht, Germany) as described previously.[84] Serum was allowed to clot and separated by centrifugation at 10,000 × *g* for 5 min at 4°C. The heart, diaphragm, TA, and triceps muscles were harvested by macrodissection. Tissues were mounted onto cork discs using O.C.T. Compound Mounting Media for Cryotomy (VWR, Lutterworth, UK) and snap frozen in dry-ice-cooled isopentane. Tissue samples were stored at −80°C prior to downstream processing. Tissues from *mdx* and WT mice were collected in parallel for the purposes of generating western blot standard curves and control sections, as appropriate.

Genomic DNA analysis

gDNA was extracted from cells using KAPA Express Extract (Kapa Biosystems, Feltham, UK) and from tissues using the DNeasy Blood & Tissue Kit (Qiagen, Manchester, UK), according to the manufacturer's instructions. PCR primers were designed to amplify a sequence that spans the targeted deletion site (Table S2). For cell culture samples, PCR was performed using KAPA 2G Fast HotStart ReadyMix (Kapa Biosystems) with an initial denaturation phase at 95°C for 3 min followed by 35 cycles of denaturing (95°C for 15 s), annealing (53°C for 15 s), and extension (72°C for 30 s). For *in vivo* samples, PCR was performed using KAPA2G Robust HotStart ReadyMix (Kapa Biosystems) with PCR conditions as above (with the annealing temperature modified to 55°C). PCR products were separated by agarose gel electrophoresis. PCR amplicons were purified and gene editing confirmed by Sanger sequencing (Source BioScience, Nottingham, UK). Individual amplicons were cloned using the TOPO TA cloning for sequencing kit (Thermo Fisher Scientific). Sanger sequencing chromatogram deconvolution was performed using the ICE online tool.[85]

qPCR

RT-qPCR was conducted following the minimum information for publication of quantitative real-time PCR experiments (MIQE) guidelines.[86] Total RNA was isolated from muscle tissues (~100 μm sections, or one-sixth of the diaphragm) using a Maxwell RSC Instrument and Maxwell RSC simplyRNA Tissue Kit (both Promega, Southampton, UK), and cDNA was reverse-transcribed using the High-Capacity cDNA Reverse Transcription Kit (Thermo Fisher Scientific) according to the manufacturer's instructions (using random primers). qPCR was carried out using TaqMan probe-based assays to determine the level of *Dmd* exon 23 skipping as a consequence of transcription from the edited *Dmd* locus. qPCR reactions were performed using TaqMan Universal PCR Master Mix (Thermo Fisher Scientific) and the StepOnePlus Real-Time PCR System (Applied Biosystems) with the following cycling conditions: 95°C for 10 min, followed by 40 cycles of 95°C for 15 s and 60°C for 1 min.

Absolute quantification was performed by comparing samples with standard curves composed of serial dilutions of the full-length and ΔExon23 *Dmd* DNA target templates (IDT, Leuven, Belgium). The degree of exon 23 excision was determined by calculating the percentage of ΔExon23 *Dmd* transcripts relative to the total (i.e., full-length + ΔExon23 *Dmd* transcripts). All primer sequences are listed in Table S2.

AAV vector genome copies were determined by absolute quantification qPCR using primers designed to amplify the SaCas9 transgene (Table S2). Samples were compared with serial dilutions of plasmid DNA in order to calculate copy numbers. To measure host genome copy numbers, a commercial TaqMan assay designed to amplify the *Actb* gene (Mm00607939_s1, Applied Biosystems) was used, and amplification results compared against a serial dilution of mouse gDNA of known mass. qPCR was performed as described above, except for SaCas9 mRNA quantification, where Power SYBR Green Master Mix (Thermo Fisher Scientific) was used.

Serum miRNA analysis

Extracellular miRNAs were analyzed by small RNA TaqMan RT-qPCR as described previously.[84,87] Briefly, RNA was extracted from 50 μL of serum using TRIzol LS (Thermo Fisher Scientific) according to manufacturer's instructions, with minor modifications. RNase-free glycogen (Roche) was used as an inert carrier to maximize RNA recovery, and a synthetic oligonucleotide spike-in control (cel-miR-39, 5'-UCACCGGGUGUAAAUCAGCUUG-3', IDT) was added at the phenolic extraction stage to enable later data normalization. RNA was re-suspended in 30 μL of nuclease-free water (Thermo Fisher Scientific) and 10 μL of each sample used for reverse transcription using the TaqMan MicroRNA Reverse Transcription Kit (Thermo Fisher Scientific) according to manufacturer's instructions. Thermal cycling was performed as described above. Data were normalized to cel-miR-39 and data analyzed using the Pfaffl method.[88] miRNA TaqMan assays are described in Table S3.

Western blot

Total protein was isolated from muscle tissues (~100 8-μm sections, or one-sixth of the diaphragm) using modified Radioimmunoprecipitation assay (RIPA) lysis buffer (10% SDS, 50 mM tris [pH 8], 150 mM NaCl, 1% NP-40, 0.5% sodium deoxycholate), supplemented with 1× cOmplete protease inhibitor cocktail (Merck, Feltham, UK). Samples were homogenized using a Precellys 24 Tissue Homogenizer (Bertin Technologies, Paris, France) (four cycles at 5,000 rpm for 30 s). Then 20 μg of total protein lysate for each sample was separated by SDS-PAGE, using NuPAGE 3%–8% Tris-Acetate 1-mm 10-well gels (Thermo Fisher Scientific) run at 150 V for 90 min in 1× NuPAGE Tris-Acetate SDS Running Buffer (Thermo Fisher Scientific). Proteins were electrotransferred onto an Immobilon-fl polyvinylidene difluoride (PVDF) membrane (Merck) at 30 V for 1 h followed by 100 V for a further hour at 4°C in 1× NuPAGE Transfer Buffer (Thermo Fisher Scientific) supplemented with 0.1 g/L of SDS (Sigma-Aldrich, MO, USA) and 10% methanol. Membranes were blocked in Intercept (PBS) Blocking Buffer (Sigma-Aldrich) for 30 min at room temperature and then incubated with primary antibodies in Intercept Blocking Buffer supplemented with 0.1% Tween 20 (Sigma-Aldrich) at 4°C overnight. Membranes were subsequently washed with PBS supplemented with 0.1% Tween 20 (PBST), and incubated with secondary antibodies (Thermo Fisher Scientific) in blocking buffer for 1 h at room temperature. After further washing in PBST, blots were developed using Clarity Western ECL Substrate (Bio-Rad, Watford, UK) and imaged using a LI-COR Biosciences Odyssey Fc instrument (LI-COR, NE, USA). Protein standards were generated by combining dystrophin-positive (WT) and dystrophin-negative (*mdx*) protein lysates in a series of ratios, and were run in parallel to facilitate dystrophin quantification in treated samples. Vinculin was used as a loading control. Details of all antibodies are shown in Table S4.

Immunofluorescence

Eight-micrometer transverse or longitudinal sections of heart, diaphragm, TA, and triceps tissues were cryosectioned, transferred to Superfrost Microscope Slides (Thermo Fisher Scientific), and air

dried prior to storage at −80°C. Sections were soaked in PBS for 10 min before incubation in blocking solution (20% fetal calf serum [FCS; Thermo Fisher Scientific], and 20% normal goat serum (NGS, MP Biomedicals, Eschwege, Germany), in PBS) for an hour at room temperature. Sections were then incubated with primary antibodies in blocking solution for 2 h at room temperature. Sections were washed three times with PBS and then incubated with fluorescent secondary antibodies in PBS for 1 h at room temperature. Slides were again washed three times with PBS and mounted with Vectashield Hard Set mounting medium with DAPI H-500 (2BScientific, Oxfordshire, UK). Where appropriate, slides were treated with the Mouse on Mouse (M.O.M.) Detection Kit (Vector Laboratories, Peterborough, UK) following manufacturer's instructions. Sections were imaged using a Leica DMIRB Inverted Modulation Contrast Microscope using the MetaMorph imaging software (Leica Biosystems, Newcastle upon Tyne, UK). Images were processed using ImageJ software. Details of all antibodies are shown in Table S4.

Long-read amplicon sequencing

gDNA was harvested from cardiac tissue of dKO mice treated with CRISPR AAVs either by IP or IV injection (n = 2 each), as well as saline-treated control mice (n = 1), using the DNeasy Blood and Tissue kit (Qiagen, Manchester, UK). The region spanning the CRISPR target site was amplified by PCR. Briefly, PCR was carried out on 100 ng of gDNA per sample using Phusion High-Fidelity DNA Polymerase (New England Biolabs, Hitchin, UK) with an initial denaturation phase (98°C for 30 s), and then 35 cycles of denaturing (98°C for 10 s), annealing (53°C for 15 s), and extension (72°C for 30 s). PCR products were purified using the GeneJET PCR purification kit (Thermo Fisher Scientific). Sample concentrations were determined using the QuantiFluor ONE dsDNA System and a Quantus Fluorometer (both Promega, Southampton, UK). Equimolar amounts of each sample were pooled, and long-read next-generation sequencing using the PacBio platform carried out as a service by Genewiz (South Plainfield, NJ, USA). PCR primers with internal 8-mer barcodes used for de-multiplexing (bold sequence) are listed in Table S2.

The barcoded circular consensus reads were demultiplexed, and asymmetric barcodes were removed using the LIMA software package v2.4.0 (https://github.com/pacificbiosciences/barcoding/). The resulting demultiplexed FASTQ files were inspected with FastQC v.0.11.9 (http://www.bioinformatics.babraham.ac.uk/projects/fastqc/) then quality filtered with BBDuk (part of the BBTools software toolkit v.38.93; https://sourceforge.net/projects/bbmap/), with the maq parameter set to Q20. Reads shorter than 400 bases were discarded, resulting in more than 20,000 high-quality reads per sample for all subsequent analyses. Sequence classification based on presence or absence of sequence features was performed with the BBDuk and BBDuk2 packages, which are designed to filter based on look-up of *k*-mers. *k*-mer length was set to 25 bases, with a minimal edit distance, allowing for accurate filtering of reads that may contain minor sequencing errors. For example, a read was classified as AAV containing if it contained any 25-nucleotide contiguous sequence from the AAV reference, with up to two mismatches or indels allowed. The *k*-mer and edit distance parameters were optimized against the untreated sample in order to minimize incorrect classification. Furthermore, at each sequence classification step, the resulting FASTQ files were aligned to the mouse chromosome X reference sequence (mm10), using the minimap2 SMRT wrapper for PacBio data: pbmm2 v1.3.0.[89] The aligned reads were inspected using IGV v2.11.2, which supports third-generation sequencing. FASTQ file intersections based on common read identifiers were generated using seqkit v2.1.0.[90] Raw sequencing data are deposited in NCBI SRA: PRJNA789451.

Statistics

Statistical analysis was carried out using GraphPad Prism 9.0 software.

Data availability

Long-read sequencing data are deposited in NCBI SRA: PRJNA789451. All other data are included in the manuscript. Raw data are available on request.

SUPPLEMENTAL INFORMATION

Supplemental information can be found online at https://doi.org/10.1016/j.omtn.2022.10.010.

ACKNOWLEDGMENTS

B.H. is supported by a doctoral studentship from the Clarendon Fund and a grant from the Medical Life Sciences Translational Fund (MLSTF) awarded to T.C.R. S.S. is supported by a Medical Research Council (MRC) Confidence in Concept grant awarded to T.C.R. and M.J.A.W. K.C. is supported by a doctoral studentship from the Clarendon Fund in partnership with the MRC, and the Juel-Jenson Scholarship from St Cross College, Oxford. N.S. is supported by a doctoral studentship funded by Wave Life Sciences. This work was funded by an MRC Programme Grant awarded to M.J.A.W. (MRN0248501).

AUTHOR CONTRIBUTIONS

T.C.R., M.S.W., and M.J.A.W. conceived the study and supervised the work. B.H., S.S., N.A., K.C., A.M.L.C.-S., and T.C.R. performed experiments and collected data. N.S. performed sequencing data analysis. B.H. and T.C.R. wrote the first draft of the manuscript. All authors contributed to the final version of the manuscript.

DECLARATION OF INTERESTS

The authors declare no competing interests.

REFERENCES

1. Hoffman, E.P., Brown, R.H., Jr., and Kunkel, L.M. (1987). Dystrophin: the protein product of the Duchenne muscular dystrophy locus. Cell *51*, 919–928.
2. Messina, S., Bitto, A., Aguennouz, M., Minutoli, L., Monici, M.C., Altavilla, D., Squadrito, F., and Vita, G. (2006). Nuclear factor kappa-B blockade reduces skeletal muscle degeneration and enhances muscle function in Mdx mice. Exp. Neurol. *198*, 234–241. https://doi.org/10.1016/j.expneurol.2005.11.021.
3. Pastoret, C., and Sebille, A. (1995). mdx mice show progressive weakness and muscle deterioration with age. J. Neurol. Sci. *129*, 97–105. https://doi.org/10.1016/0022-510x(94)00276-t.

4. Yokota, T., Lu, Q.-L., Morgan, J.E., Davies, K.E., Fisher, R., Takeda, S., and Partridge, T.A. (2006). Expansion of revertant fibers in dystrophic mdx muscles reflects activity of muscle precursor cells and serves as an index of muscle regeneration. J. Cell Sci. *119*, 2679–2687. https://doi.org/10.1242/jcs.03000.

5. Szabo, S.M., Salhany, R.M., Deighton, A., Harwood, M., Mah, J., and Gooch, K.L. (2021). The clinical course of Duchenne muscular dystrophy in the corticosteroid treatment era: a systematic literature review. Orphanet J. Rare Dis. *16*, 237. https://doi.org/10.1186/s13023-021-01862-w.

6. Moriuchi, T., Kagawa, N., Mukoyama, M., and Hizawa, K. (1993). Autopsy analyses of the muscular dystrophies. Tokushima J. Exp. Med. *40*, 83–93.

7. Chiang, D.Y., Allen, H.D., Kim, J.J., Valdes, S.O., Wang, Y., Pignatelli, R.H., Lotze, T.E., and Miyake, C.Y. (2016). Relation of cardiac dysfunction to rhythm abnormalities in patients with Duchenne or becker muscular dystrophies. Am. J. Cardiol. *117*, 1349–1354. https://doi.org/10.1016/j.amjcard.2016.01.031.

8. Ishikawa, Y., Miura, T., Ishikawa, Y., Aoyagi, T., Ogata, H., Hamada, S., and Minami, R. (2011). Duchenne muscular dystrophy: survival by cardio-respiratory interventions. Neuromuscul. Disord. *21*, 47–51. https://doi.org/10.1016/j.nmd.2010.09.006.

9. Anthony, K., Cirak, S., Torelli, S., Tasca, G., Feng, L., Arechavala-Gomeza, V., Armaroli, A., Guglieri, M., Straathof, C.S., Verschuuren, J.J., et al. (2011). Dystrophin quantification and clinical correlations in Becker muscular dystrophy: implications for clinical trials. Brain *134*, 3547–3559. https://doi.org/10.1093/brain/awr291.

10. Monaco, A.P., Bertelson, C.J., Liechti-Gallati, S., Moser, H., and Kunkel, L.M. (1988). An explanation for the phenotypic differences between patients bearing partial deletions of the DMD locus. Genomics *2*, 90–95.

11. England, S.B., Nicholson, L.V., Johnson, M.A., Forrest, S.M., Love, D.R., Zubrzycka-Gaarn, E.E., Bulman, D.E., Harris, J.B., and Davies, K.E. (1990). Very mild muscular dystrophy associated with the deletion of 46% of dystrophin. Nature *343*, 180–182. https://doi.org/10.1038/343180a0.

12. Matsumura, K., Burghes, A.H., Mora, M., Tomé, F.M., Morandi, L., Cornello, F., Leturcq, F., Jeanpierre, M., Kaplan, J.C., and Reinert, P. (1994). Immunohistochemical analysis of dystrophin-associated proteins in Becker/Duchenne muscular dystrophy with huge in-frame deletions in the NH2-terminal and rod domains of dystrophin. J. Clin. Invest. *93*, 99–105. https://doi.org/10.1172/JCI116989.

13. Koenig, M., Beggs, A.H., Moyer, M., Scherpf, S., Heindrich, K., Bettecken, T., Meng, G., Müller, C.R., Lindlöf, M., Kaariainen, H., et al. (1989). The molecular basis for Duchenne versus Becker muscular dystrophy: correlation of severity with type of deletion. Am. J. Hum. Genet. *45*, 498–506.

14. Emery, A.E. (1991). Population frequencies of inherited neuromuscular diseases–a world survey. Neuromuscul. Disord. *1*, 19–29.

15. Koenig, M., Hoffman, E.P., Bertelson, C.J., Monaco, A.P., Feener, C., and Kunkel, L.M. (1987). Complete cloning of the Duchenne muscular dystrophy (DMD) cDNA and preliminary genomic organization of the DMD gene in normal and affected individuals. Cell *50*, 509–517.

16. Aartsma-Rus, A., Fokkema, I., Verschuuren, J., Ginjaar, I., van Deutekom, J., van Ommen, G.-J., and den Dunnen, J.T. (2009). Theoretic applicability of antisense-mediated exon skipping for Duchenne muscular dystrophy mutations. Hum. Mutat. *30*, 293–299. https://doi.org/10.1002/humu.20918.

17. Roberts, T.C., Langer, R., and Wood, M.J.A. (2020). Advances in oligonucleotide drug delivery. Nat. Rev. Drug Discov. *19*, 673–694. https://doi.org/10.1038/s41573-020-0075-7.

18. Charleston, J.S., Schnell, F.J., Dworzak, J., Donoghue, C., Lewis, S., Chen, L., Young, G.D., Milici, A.J., Voss, J., DeAlwis, U., et al. (2018). Eteplirsen treatment for Duchenne muscular dystrophy: exon skipping and dystrophin production. Neurology *90*, e2146–e2154. https://doi.org/10.1212/WNL.0000000000005680.

19. Jinek, M., Chylinski, K., Fonfara, I., Hauer, M., Doudna, J.A., and Charpentier, E. (2012). A programmable dual-RNA–guided DNA endonuclease in adaptive bacterial immunity. Science *337*, 816–821. https://doi.org/10.1126/science.1225829.

20. Cong, L., Ran, F.A., Cox, D., Lin, S., Barretto, R., Habib, N., Hsu, P.D., Wu, X., Jiang, W., Marraffini, L.A., and Zhang, F. (2013). Multiplex genome engineering using CRISPR/Cas systems. Science *339*, 819–823. https://doi.org/10.1126/science.1231143.

21. Mali, P., Yang, L., Esvelt, K.M., Aach, J., Guell, M., DiCarlo, J.E., Norville, J.E., and Church, G.M. (2013). RNA-guided human genome engineering via Cas9. Science *339*, 823–826. https://doi.org/10.1126/science.1232033.

22. Heyer, W.-D., Ehmsen, K.T., and Liu, J. (2010). Regulation of homologous recombination in eukaryotes. Annu. Rev. Genet. *44*, 113–139. https://doi.org/10.1146/annurev-genet-051710-150955.

23. Nelson, C.E., Hakim, C.H., Ousterout, D.G., Thakore, P.I., Moreb, E.A., Castellanos Rivera, R.M., Madhavan, S., Pan, X., Ran, F.A., Yan, W.X., et al. (2016). In vivo genome editing improves muscle function in a mouse model of Duchenne muscular dystrophy. Science *351*, 403–407. https://doi.org/10.1126/science.aad5143.

24. Tabebordbar, M., Zhu, K., Cheng, J.K.W., Chew, W.L., Widrick, J.J., Yan, W.X., Maesner, C., Wu, E.Y., Xiao, R., Ran, F.A., et al. (2016). In vivo gene editing in dystrophic mouse muscle and muscle stem cells. Science *351*, 407–411. https://doi.org/10.1126/science.aad5177.

25. Nelson, C.E., Wu, Y., Gemberling, M.P., Oliver, M.L., Waller, M.A., Bohning, J.D., Robinson-Hamm, J.N., Bulaklak, K., Castellanos Rivera, R.M., Collier, J.H., et al. (2019). Long-term evaluation of AAV-CRISPR genome editing for Duchenne muscular dystrophy. Nat. Med. *25*, 427–432. https://doi.org/10.1038/s41591-019-0344-3.

26. Bengtsson, N.E., Hall, J.K., Odom, G.L., Phelps, M.P., Andrus, C.R., Hawkins, R.D., Hauschka, S.D., Chamberlain, J.R., and Chamberlain, J.S. (2017). Muscle-specific CRISPR/Cas9 dystrophin gene editing ameliorates pathophysiology in a mouse model for Duchenne muscular dystrophy. Nat. Commun. *8*, 14454. https://doi.org/10.1038/ncomms14454.

27. Long, C., Amoasii, L., Mireault, A.A., McAnally, J.R., Li, H., Sanchez-Ortiz, E., Bhattacharyya, S., Shelton, J.M., Bassel-Duby, R., and Olson, E.N. (2016). Postnatal genome editing partially restores dystrophin expression in a mouse model of muscular dystrophy. Science *351*, 400–403. https://doi.org/10.1126/science.aad5725.

28. Moretti, A., Fonteyne, L., Giesert, F., Hoppmann, P., Meier, A.B., Bozoglu, T., Baehr, A., Schneider, C.M., Sinnecker, D., Klett, K., et al. (2020). Somatic gene editing ameliorates skeletal and cardiac muscle failure in pig and human models of Duchenne muscular dystrophy. Nat. Med. *26*, 207–214. https://doi.org/10.1038/s41591-019-0738-2.

29. Amoasii, L., Long, C., Li, H., Mireault, A.A., Shelton, J.M., Sanchez-Ortiz, E., McAnally, J.R., Bhattacharyya, S., Schmidt, F., Grimm, D., et al. (2017). Single-cut genome editing restores dystrophin expression in a new mouse model of muscular dystrophy. Sci. Transl. Med. *9*, eaan8081. https://doi.org/10.1126/scitranslmed.aan8081.

30. Amoasii, L., Hildyard, J.C.W., Li, H., Sanchez-Ortiz, E., Mireault, A., Caballero, D., Harron, R., Stathopoulou, T.-R., Massey, C., Shelton, J.M., et al. (2018). Gene editing restores dystrophin expression in a canine model of Duchenne muscular dystrophy. Science *362*, 86–91. https://doi.org/10.1126/science.aau1549.

31. Hakim, C.H., Wasala, N.B., Nelson, C.E., Wasala, L.P., Yue, Y., Louderman, J.A., Lessa, T.B., Dai, A., Zhang, K., Jenkins, G.J., et al. (2018). AAV CRISPR editing rescues cardiac and muscle function for 18 months in dystrophic mice. JCI Insight *3*, e124297. https://doi.org/10.1172/jci.insight.124297.

32. Min, Y.-L., Li, H., Rodriguez-Caycedo, C., Mireault, A.A., Huang, J., Shelton, J.M., McAnally, J.R., Amoasii, L., Mammen, P.P.A., Bassel-Duby, R., and Olson, E.N. (2019). CRISPR-Cas9 corrects Duchenne muscular dystrophy exon 44 deletion mutations in mice and human cells. Sci. Adv. *5*, eaav4324. https://doi.org/10.1126/sciadv.aav4324.

33. Zhang, Y., Li, H., Min, Y.-L., Sanchez-Ortiz, E., Huang, J., Mireault, A.A., Shelton, J.M., Kim, J., Mammen, P.P.A., Bassel-Duby, R., and Olson, E.N. (2020). Enhanced CRISPR-Cas9 correction of Duchenne muscular dystrophy in mice by a self-complementary AAV delivery system. Sci. Adv. *6*, eaay6812. https://doi.org/10.1126/sciadv.aay6812.

34. Hanson, B., Wood, M.J.A., and Roberts, T.C. (2021). Molecular correction of Duchenne muscular dystrophy by splice modulation and gene editing. RNA Biol. *18*, 1048–1062. https://doi.org/10.1080/15476286.2021.1874161.

35. Senís, E., Fatouros, C., Große, S., Wiedtke, E., Niopek, D., Mueller, A.-K., Börner, K., and Grimm, D. (2014). CRISPR/Cas9-mediated genome engineering: an adeno-associated viral (AAV) vector toolbox. Biotechnol. J. *9*, 1402–1412. https://doi.org/10.1002/biot.201400046.

36. Gruntman, A.M., Bish, L.T., Mueller, C., Sweeney, H.L., Flotte, T.R., and Gao, G. (2013). Gene transfer in skeletal and cardiac muscle using recombinant adeno-associated virus. Curr. Protoc. Microbiol. *14*. Unit 14D.3. https://doi.org/10.1002/9780471729259.mc14d03s28.

37. Qiao, C., Koo, T., Li, J., Xiao, X., and Dickson, J.G. (2011). Gene therapy in skeletal muscle mediated by adeno-associated virus vectors. Methods Mol. Biol. *807*, 119–140. https://doi.org/10.1007/978-1-61779-370-7_5.

38. Kotterman, M.A., and Schaffer, D.V. (2014). Engineering adeno-associated viruses for clinical gene therapy. Nat. Rev. Genet. *15*, 445–451. https://doi.org/10.1038/nrg3742.

39. Nathwani, A.C., Rosales, C., McIntosh, J., Rastegarlari, G., Nathwani, D., Raj, D., Nawathe, S., Waddington, S.N., Bronson, R., Jackson, S., et al. (2011). Long-term safety and efficacy following systemic administration of a self-complementary AAV vector encoding human FIX pseudotyped with serotype 5 and 8 capsid proteins. Mol. Ther. *19*, 876–885. https://doi.org/10.1038/mt.2010.274.

40. Esvelt, K.M., Mali, P., Braff, J.L., Moosburner, M., Yaung, S.J., and Church, G.M. (2013). Orthogonal Cas9 proteins for RNA-guided gene regulation and editing. Nat. Methods *10*, 1116–1121. https://doi.org/10.1038/nmeth.2681.

41. Ran, F.A., Cong, L., Yan, W.X., Scott, D.A., Gootenberg, J.S., Kriz, A.J., Zetsche, B., Shalem, O., Wu, X., Makarova, K.S., et al. (2015). In vivo genome editing using Staphylococcus aureus Cas9. Nature *520*, 186–191. https://doi.org/10.1038/nature14299.

42. Chamberlain, J.S., Metzger, J., Reyes, M., Townsend, D., and Faulkner, J.A. (2007). Dystrophin-deficient mdx mice display a reduced life span and are susceptible to spontaneous rhabdomyosarcoma. Faseb. J. *21*, 2195–2204. https://doi.org/10.1096/fj.06-7353com.

43. Coenen-Stass, A.M.L., Betts, C.A., Lee, Y.F., Mäger, I., Turunen, M.P., El Andaloussi, S., Morgan, J.E., Wood, M.J.A., and Roberts, T.C. (2016). Selective release of muscle-specific, extracellular microRNAs during myogenic differentiation. Hum. Mol. Genet. *25*, 3960–3974. https://doi.org/10.1093/hmg/ddw237.

44. Betts, C.A., Saleh, A.F., Carr, C.A., Hammond, S.M., Coenen-Stass, A.M.L., Godfrey, C., McClorey, G., Varela, M.A., Roberts, T.C., Clarke, K., et al. (2015). Prevention of exercised induced cardiomyopathy following Pip-PMO treatment in dystrophic mdx mice. Sci. Rep. *5*, 8986. https://doi.org/10.1038/srep08986.

45. van Westering, T.L.E., Johansson, H.J., Hanson, B., Coenen-Stass, A.M.L., Lomonosova, Y., Tanihata, J., Motohashi, N., Yokota, T., Takeda, S., Lehtiö, J., et al. (2020). Mutation-independent proteomic signatures of pathological progression in murine models of Duchenne muscular dystrophy. Mol. Cell. Proteomics *19*, 2047–2068. https://doi.org/10.1074/mcp.RA120.002345.

46. Deconinck, A.E., Rafael, J.A., Skinner, J.A., Brown, S.C., Potter, A.C., Metzinger, L., Watt, D.J., Dickson, J.G., Tinsley, J.M., and Davies, K.E. (1997). Utrophin-dystrophin-deficient mice as a model for Duchenne muscular dystrophy. Cell *90*, 717–727.

47. Grady, R.M., Teng, H., Nichol, M.C., Cunningham, J.C., Wilkinson, R.S., and Sanes, J.R. (1997). Skeletal and cardiac myopathies in mice lacking utrophin and dystrophin: a model for Duchenne muscular dystrophy. Cell *90*, 729–738. https://doi.org/10.1016/s0092-8674(00)80533-4.

48. Goyenvalle, A., Babbs, A., Powell, D., Kole, R., Fletcher, S., Wilton, S.D., and Davies, K.E. (2010). Prevention of dystrophic pathology in severely affected dystrophin/utrophin-deficient mice by morpholino-oligomer-mediated exon-skipping. Mol. Ther. *18*, 198–205. https://doi.org/10.1038/mt.2009.248.

49. Goyenvalle, A., Babbs, A., Wright, J., Wilkins, V., Powell, D., Garcia, L., and Davies, K.E. (2012). Rescue of severely affected dystrophin/utrophin-deficient mice through scAAV-U7snRNA-mediated exon skipping. Hum. Mol. Genet. *21*, 2559–2571. https://doi.org/10.1093/hmg/dds082.

50. Wu, Z., Yang, H., and Colosi, P. (2010). Effect of genome size on AAV vector packaging. Mol. Ther. *18*, 80–86. https://doi.org/10.1038/mt.2009.255.

51. Komaki, H., Nagata, T., Saito, T., Masuda, S., Takeshita, E., Sasaki, M., Tachimori, H., Nakamura, H., Aoki, Y., and Takeda, S. (2018). Systemic administration of the antisense oligonucleotide NS-065/NCNP-01 for skipping of exon 53 in patients with Duchenne muscular dystrophy. Sci. Transl. Med. *10*, eaan0713. https://doi.org/10.1126/scitranslmed.aan0713.

52. van Westering, T.L.E., Lomonosova, Y., Coenen-Stass, A.M.L., Betts, C.A., Bhomra, A., Hulsker, M., Clark, L.E., McClorey, G., Aartsma-Rus, A., van Putten, M., et al. (2020). Uniform sarcolemmal dystrophin expression is required to prevent extracellular microRNA release and improve dystrophic pathology. J. Cachexia Sarcopenia Muscle *11*, 578–593. https://doi.org/10.1002/jcsm.12506.

53. Ervasti, J.M., and Campbell, K.P. (1991). Membrane organization of the dystrophin-glycoprotein complex. Cell *66*, 1121–1131.

54. Scaglioni, D., Catapano, F., Ellis, M., Torelli, S., Chambers, D., Feng, L., Beck, M., Sewry, C., Monforte, M., Harriman, S., et al. (2021). The administration of antisense oligonucleotide golodirsen reduces pathological regeneration in patients with Duchenne muscular dystrophy. Acta Neuropathol. Commun. *9*, 7. https://doi.org/10.1186/s40478-020-01106-1.

55. El Refaey, M., Xu, L., Gao, Y., Canan, B.D., Adesanya, T.M.A., Warner, S.C., Akagi, K., Symer, D.E., Mohler, P.J., Ma, J., et al. (2017). In vivo genome editing restores dystrophin expression and cardiac function in dystrophic mice. Circ. Res. *121*, 923–929. https://doi.org/10.1161/CIRCRESAHA.117.310996.

56. Wu, B., Cloer, C., Lu, P., Milazi, S., Shaban, M., Shah, S.N., Marston-Poe, L., Moulton, H.M., and Lu, Q.L. (2014). Exon skipping restores dystrophin expression, but fails to prevent disease progression in later stage dystrophic dko mice. Gene Ther. *21*, 785–793. https://doi.org/10.1038/gt.2014.53.

57. Verhaart, I.E.C., van Vliet-van den Dool, L., Sipkens, J.A., de Kimpe, S.J., Kolfschoten, I.G.M., van Deutekom, J.C.T., Liefaard, L., Ridings, J.E., Hood, S.R., and Aartsma-Rus, A. (2014). The dynamics of compound, transcript, and protein effects after treatment with 2OMePS antisense oligonucleotides in mdx mice. Mol. Ther. Nucleic Acids *3*, e148. https://doi.org/10.1038/mtna.2014.1.

58. Wu, B., Lu, P., Cloer, C., Shaban, M., Grewal, S., Milazi, S., Shah, S.N., Moulton, H.M., and Lu, Q.L. (2012). Long-term rescue of dystrophin expression and improvement in muscle pathology and function in dystrophic mdx mice by peptide-conjugated morpholino. Am. J. Pathol. *181*, 392–400. https://doi.org/10.1016/j.ajpath.2012.04.006.

59. Long, C., McAnally, J.R., Shelton, J.M., Mireault, A.A., Bassel-Duby, R., and Olson, E.N. (2014). Prevention of muscular dystrophy in mice by CRISPR/Cas9–mediated editing of germline DNA. Science *345*, 1184–1188. https://doi.org/10.1126/science.1254445.

60. Neri, M., Torelli, S., Brown, S., Ugo, I., Sabatelli, P., Merlini, L., Spitali, P., Rimessi, P., Gualandi, F., Sewry, C., et al. (2007). Dystrophin levels as low as 30% are sufficient to avoid muscular dystrophy in the human. Neuromuscul. Disord. *17*, 913–918. https://doi.org/10.1016/j.nmd.2007.07.005.

61. Godfrey, C., Muses, S., McClorey, G., Wells, K.E., Coursindel, T., Terry, R.L., Betts, C., Hammond, S., O'Donovan, L., Hildyard, J., et al. (2015). How much dystrophin is enough: the physiological consequences of different levels of dystrophin in the mdx mouse. Hum. Mol. Genet. *24*, 4225–4237. https://doi.org/10.1093/hmg/ddv155.

62. Wells, D.J., Wells, K.E., Asante, E.A., Turner, G., Sunada, Y., Campbell, K.P., Walsh, F.S., and Dickson, G. (1995). Expression of human full-length and minidystrophin in transgenic mdx mice: implications for gene therapy of Duchenne muscular dystrophy. Hum. Mol. Genet. *4*, 1245–1250. https://doi.org/10.1093/hmg/4.8.1245.

63. (2020). High-dose AAV gene therapy deaths. Nat. Biotechnol. *38*, 910. https://doi.org/10.1038/s41587-020-0642-9.

64. Lysogene Confirms Child's Death in Phase II/III Gene Therapy Trial. https://www.genengnews.com/news/lysogene-confirms-childs-death-in-phase-ii-iii-gene-therapy-trial/.

65. Vinluan, F. (2021). Patient Death Prompts FDA Halt on Pfizer's Duchenne Gene Therapy Study. MedCity News. https://medcitynews.com/2021/12/patient-death-prompts-fda-halt-on-pfizers-duchenne-gene-therapy-study/.

66. Reuters. (2022). Novartis Reports Zolgensma Caused Two Deaths from Liver Failure (Reuters).

67. McCarty, D.M., Fu, H., Monahan, P.E., Toulson, C.E., Naik, P., and Samulski, R.J. (2003). Adeno-associated virus terminal repeat (TR) mutant generates self-complementary vectors to overcome the rate-limiting step to transduction in vivo. Gene Ther. *10*, 2112–2118. https://doi.org/10.1038/sj.gt.3302134.

68. Wang, Z., Ma, H.-I., Li, J., Sun, L., Zhang, J., and Xiao, X. (2003). Rapid and highly efficient transduction by double-stranded adeno-associated virus vectors in vitro and in vivo. Gene Ther. *10*, 2105–2111. https://doi.org/10.1038/sj.gt.3302133.

69. Marsh, S., Hanson, B., Wood, M.J.A., Varela, M.A., and Roberts, T.C. (2020). Application of CRISPR-Cas9-mediated genome editing for the treatment of myotonic

dystrophy type 1. Mol. Ther. *28*, 2527–2539. https://doi.org/10.1016/j.ymthe.2020.10.005.

70. Hall, Z.W., and Ralston, E. (1989). Nuclear domains in muscle cells. Cell *59*, 771–772. https://doi.org/10.1016/0092-8674(89)90597-7.

71. Petrof, B.J., Shrager, J.B., Stedman, H.H., Kelly, A.M., and Sweeney, H.L. (1993). Dystrophin protects the sarcolemma from stresses developed during muscle contraction. Proc. Natl. Acad. Sci. USA *90*, 3710–3714.

72. van Putten, M., Hulsker, M., Nadarajah, V.D., van Heiningen, S.H., van Huizen, E., van Iterson, M., Admiraal, P., Messemaker, T., den Dunnen, J.T., 't Hoen, P.A.C., and Aartsma-Rus, A. (2012). The effects of low levels of dystrophin on mouse muscle function and pathology. PLoS One *7*, e31937. https://doi.org/10.1371/journal.pone.0031937.

73. Chwalenia, K., Oieni, J., Zemła, J., Lekka, M., Ahlskog, N., Coenen-Stass, A.M.L., McClorey, G., Wood, M.J.A., Lomonosova, Y., and Roberts, T.C. (2022). Exon skipping induces uniform dystrophin rescue with dose-dependent restoration of serum miRNA biomarkers and muscle biophysical properties. Mol. Ther. Nucleic Acids *29*, 955–968. https://doi.org/10.1016/j.omtn.2022.08.033.

74. van Putten, M., Hulsker, M., Young, C., Nadarajah, V.D., Heemskerk, H., van der Weerd, L., 't Hoen, P.A.C., van Ommen, G.-J.B., and Aartsma-Rus, A.M. (2013). Low dystrophin levels increase survival and improve muscle pathology and function in dystrophin/utrophin double-knockout mice. Faseb. J. *27*, 2484–2495. https://doi.org/10.1096/fj.12-224170.

75. Hoffman, E.P., Fischbeck, K.H., Brown, R.H., Johnson, M., Medori, R., Loike, J.D., Harris, J.B., Waterston, R., Brooke, M., and Specht, L. (1988). Characterization of dystrophin in muscle-biopsy specimens from patients with Duchenne's or Becker's muscular dystrophy. N. Engl. J. Med. *318*, 1363–1368. https://doi.org/10.1056/NEJM198805263182104.

76. Wang, Y., Hao, L., Wang, H., Santostefano, K., Thapa, A., Cleary, J., Li, H., Guo, X., Terada, N., Ashizawa, T., and Xia, G. (2018). Therapeutic genome editing for myotonic dystrophy type 1 using CRISPR/Cas9. Mol. Ther. *26*, 2617–2630. https://doi.org/10.1016/j.ymthe.2018.09.003.

77. Hanlon, K.S., Kleinstiver, B.P., Garcia, S.P., Zaborowski, M.P., Volak, A., Spirig, S.E., Muller, A., Sousa, A.A., Tsai, S.Q., Bengtsson, N.E., et al. (2019). High levels of AAV vector integration into CRISPR-induced DNA breaks. Nat. Commun. *10*, 4439. https://doi.org/10.1038/s41467-019-12449-2.

78. Zhang, Y., Long, C., Li, H., McAnally, J.R., Baskin, K.K., Shelton, J.M., Bassel-Duby, R., and Olson, E.N. (2017). CRISPR-Cpf1 correction of muscular dystrophy mutations in human cardiomyocytes and mice. Sci. Adv. *3*, e1602814. https://doi.org/10.1126/sciadv.1602814.

79. Arnett, A.L., Konieczny, P., Ramos, J.N., Hall, J., Odom, G., Yablonka-Reuveni, Z., Chamberlain, J.R., and Chamberlain, J.S. (2014). Adeno-associated viral (AAV) vectors do not efficiently target muscle satellite cells. Mol. Ther. Methods Clin. Dev. *1*, 14038. https://doi.org/10.1038/mtm.2014.38.

80. Hakim, C.H., Kumar, S.R.P., Pérez-López, D.O., Wasala, N.B., Zhang, D., Yue, Y., Teixeira, J., Pan, X., Zhang, K., Million, E.D., et al. (2021). Cas9-specific immune responses compromise local and systemic AAV CRISPR therapy in multiple dystrophic canine models. Nat. Commun. *12*, 6769. https://doi.org/10.1038/s41467-021-26830-7.

81. Simhadri, V.L., McGill, J., McMahon, S., Wang, J., Jiang, H., and Sauna, Z.E. (2018). Prevalence of pre-existing antibodies to CRISPR-associated nuclease Cas9 in the USA population. Mol. Ther. Methods Clin. Dev. *10*, 105–112. https://doi.org/10.1016/j.omtm.2018.06.006.

82. Wagner, D.L., Amini, L., Wendering, D.J., Burkhardt, L.-M., Akyüz, L., Reinke, P., Volk, H.-D., and Schmueck-Henneresse, M. (2019). High prevalence of Streptococcus pyogenes Cas9-reactive T cells within the adult human population. Nat. Med. *25*, 242–248. https://doi.org/10.1038/s41591-018-0204-6.

83. Engler, C., Kandzia, R., and Marillonnet, S. (2008). A one pot, one step, precision cloning method with high throughput capability. PLoS One *3*, e3647. https://doi.org/10.1371/journal.pone.0003647.

84. Roberts, T.C., Coenen-Stass, A.M.L., Betts, C.A., and Wood, M.J.A. (2014). Detection and quantification of extracellular microRNAs in murine biofluids. Biol. Proced. Online *16*, 5. https://doi.org/10.1186/1480-9222-16-5.

85. Conant, D., Hsiau, T., Rossi, N., Oki, J., Maures, T., Waite, K., Yang, J., Joshi, S., Kelso, R., Holden, K., et al. (2022). Inference of CRISPR edits from Sanger trace data. CRISPR J. *5*, 123–130. https://doi.org/10.1089/crispr.2021.0113.

86. Bustin, S.A., Benes, V., Garson, J.A., Hellemans, J., Huggett, J., Kubista, M., Mueller, R., Nolan, T., Pfaffl, M.W., Shipley, G.L., et al. (2009). The MIQE guidelines: minimum information for publication of quantitative real-time PCR experiments. Clin. Chem. *55*, 611–622. https://doi.org/10.1373/clinchem.2008.112797.

87. Roberts, T.C., Coenen-Stass, A.M.L., and Wood, M.J.A. (2014). Assessment of RT-qPCR normalization strategies for accurate quantification of extracellular microRNAs in murine serum. PLoS One *9*, e89237. https://doi.org/10.1371/journal.pone.0089237.

88. Pfaffl, M.W. (2001). A new mathematical model for relative quantification in real-time RT-PCR. Nucleic Acids Res. *29*, e45.

89. Li, H. (2018). Minimap2: pairwise alignment for nucleotide sequences. Bioinformatics *34*, 3094–3100. https://doi.org/10.1093/bioinformatics/bty191.

90. Shen, W., Le, S., Li, Y., and Hu, F. (2016). SeqKit: a cross-platform and ultrafast toolkit for FASTA/Q file manipulation. PLoS One *11*, e0163962. https://doi.org/10.1371/journal.pone.0163962.

OMTN, Volume *30*

Supplemental information

Non-uniform dystrophin re-expression after CRISPR-mediated exon excision in the dystrophin/ utrophin double-knockout mouse model of DMD

Britt Hanson, Sofia Stenler, Nina Ahlskog, Katarzyna Chwalenia, Nenad Svrzikapa, Anna M.L. Coenen-Stass, Marc S. Weinberg, Matthew J.A. Wood, and Thomas C. Roberts

Supplemental Information

Table S1

Targets for sgRNA sequences.

ID	Target Sequence (5' to 3')
Dmd-ex23-sgRNA-1	TTATTTACCTTCTTCTTGAT
Dmd-ex23-sgRNA-2	CAAATATGCGTGTTAGTGTA
Dmd-ex23-sgRNA-3	AGTCCTTCAAAGATATTGAT
Dmd-ex23-sgRNA-4	CAAAAGCCAAATCTATTTCA

Table S2

List of primer sequences used in this study.

Exon skipping RT-qPCR assay probes include 5′ terminal fluorophores (either FAM or HEX), 3′-terminal Iowa Black fluorescence quencher, and contain internal ZEN modifications. Barcode regions in the primers used for long-read amplicon sequencing are highlighted in bold.

ID	Sequence (5' to 3')
**gDNA Analysis**	
**Dmd-check-Fwd**	GCCTAAATGTCTTAATAATGTTTCAC
**Dmd-check-Rev**	GCTGTGAGCTAAATCATATCTACA
**Exon Skipping RT-qPCR**	
**qExon22-24-Fwd**	CTGAATATGAAATAATGGAGGAGAGACTCG
**qExon22-24-Rev**	CTTCAGCCATCCATTTCTGTAAGGT
**qExon22-24-Probe**	/5FAM/ATGTGATTC/ZEN/TGTAATTTCC/3IABkFQ/
**qExon23-24-Fwd**	CAGGCCATTCCTCTTTCAGG
**qExon23-24-Rev**	GAAACTTTCCTCCCAGTTGGT
**qExon23-24-Probe**	/5HEX/TCAACTTCA/ZEN/GCCATCCATTTCTGTAAGGT/3IABkFQ/
**DNA qPCR**	
**qSaCas9-Fwd**	CCAACGCCGATTTCATCTTC
**qSaCas9-Rev**	GATCTCTTTGTACTCCTGCTCG
**Long-read amplicon sequencing**	
**A701_Dmd_Check_Fwd**	**ATCACGAC**GCCTAAATGTCTTAATAATGTTTCAC
**A501_Dmd_Check_Rev**	**AAGGTTCA**GCTGTGAGCTAAATCATATCTACA
**A702_Dmd_Check_Fwd**	**ACAGTGGT**GCCTAAATGTCTTAATAATGTTTCAC
**A502_Dmd_Check_Rev**	**ACTTAGCA**GCTGTGAGCTAAATCATATCTACA
**A703_Dmd_Check_Fwd**	**CAGATCCA**GCCTAAATGTCTTAATAATGTTTCAC
**A503_Dmd_Check_Rev**	**AGAGAACA**GCTGTGAGCTAAATCATATCTACA
**A704_Dmd_Check_Fwd**	**ACAAACGG**GCCTAAATGTCTTAATAATGTTTCAC
**A504_Dmd_Check_Rev**	**GTGTCTTA**GCTGTGAGCTAAATCATATCTACA
**A705_Dmd_Check_Fwd**	**ACCCAGCA**GCCTAAATGTCTTAATAATGTTTCAC
**A505_Dmd_Check_Rev**	**TCGATTAG**GCTGTGAGCTAAATCATATCTACA

Table S3

List of TaqMan assays used in this study.

All products were obtained from Thermo Fisher Scientific.

Target	Product ID	Detection Channel
**Small RNA TaqMan RT-qPCR**		
**mmu-miR-1a-3p**	002222	FAM
**mmu-miR-133a-3p**	002246	FAM
**mmu-miR-206-3p**	000510	FAM
**mmu-miR-483-3p**	002560	FAM
**cel-miR-39**	000200	FAM
**DNA qPCR**		
Actb	Mm00607939_s1*	VIC

Table S4

Antibodies used in this study.

Target protein	Host	Product ID	Manufacturer	Dilution
**Western Blot**				
**Dystrophin (DMD)**	Mouse mAb	NCL-DYS1	Leica Biosystems	1:100
**Vinculin (VCL)**	Mouse mAb	V9131	Sigma-Aldrich/Merck	1:200
**Anti-mouse IgG, HRP-linked**	Horse	7076	Cell Signaling Technology	1:5,000
**Immunofluorescence**				
**Dystrophin (DMD)**	Rabbit pAb	ab15277	Abcam	1:1,000
**β-Dystroglycan (DAG1)**	Mouse mAb	NCL-b-DG	Leica Biosystems	1:100
**α-Dystrobrevin (DTNA1)**	Rabbit pAb	Alpha-1CTFP	In house (gift from K.E. Davies)	1:100
**nNOS (NOS1)**	Rabbit mAb	Ab76067	Abcam	1:100
**Laminin subunit alpha 2 (LAMA2)**	Rat mAb	L0663 (clone: 4H8-2)	Sigma-Aldrich/Merck	1:1,000
**Anti-rabbit IgG Alexa Fluor 594**	Goat	ab150080	Abcam	1:500
**Anti-rat IgG Alexa Fluor 488**	Goat	ab150157	Abcam	1:500

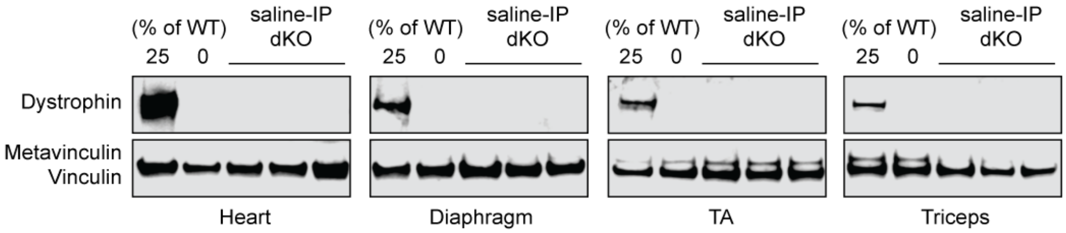

Figure S1

Dystrophin protein is undetectable by western blot in saline-treated dKO mouse tissues.

Western blot for dystrophin in 20 μg of total protein obtained from the heart, diaphragm, TA, and triceps muscles. Positive control samples contain a mixture of 25% WT (C57/BL10) with 75% dystrophin-deficient *mdx* protein. Negative control samples contain *mdx* protein only. The remaining samples consist of dKO animals treated with saline by IP injection. Vinculin was included as loading control.

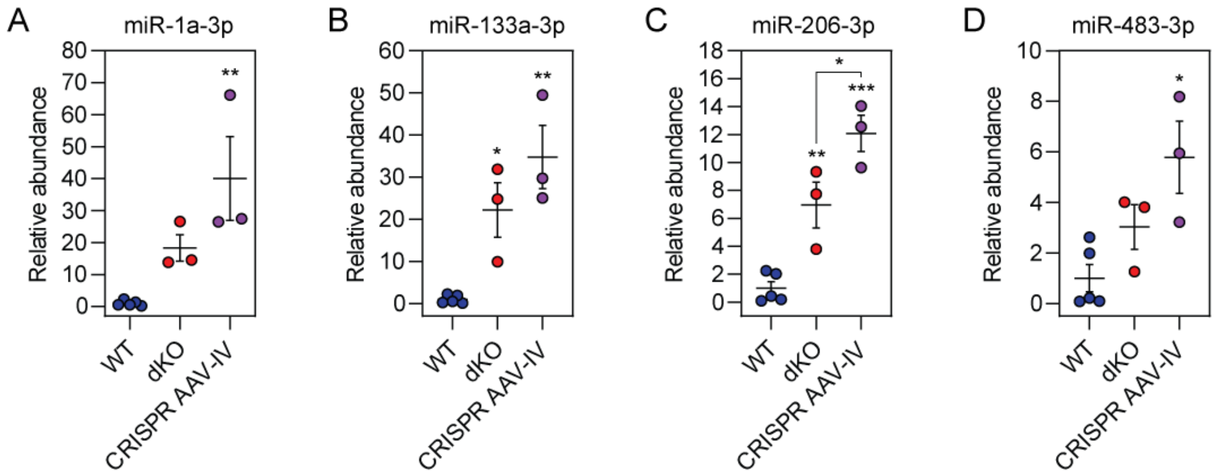

Figure S2

miRNA biomarkers are not restored to wild-type levels in CRISPR AAV-treated dKO mouse serum.

dKO mice were treated by intravenous (IV) injection via the facial vein at postnatal day two (P2) with 1×10^{11} vg of SaCas9-AAV and 5×10^{11} vg of dual sgRNA-AAV and sacrificed at the humane end point. Serum was harvested from the animals immediately postmortem (*n*=3) and RNA extracted. Serum RNA was analyzed for (**A**) miR-1a-3p, (**B**) miR-133a-3p, (**C**) miR-206-3p, and (**D**) miR-483-3p by small RNA TaqMan RT-qPCR. Serum from un-injected wild-type (WT) (*n*=5) and dKO (*n*=3) animals served as controls (10-weeks-old). Values are mean±SEM. Statistically significant differences were tested by one-way ANOVA with Bonferroni *post hoc* test, *$P<0.05$, **$P<0.01$, ***$P<0.001$.

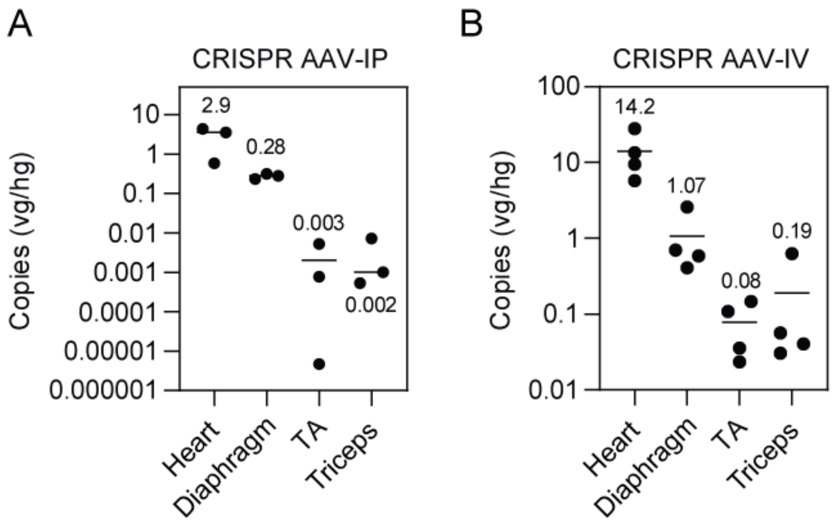

Figure S3

Biodistribution of SaCas9 vector genomes.

DNA was extracted from CRISPR AAV-treated dKO mice for (**A**) intraperitoneal (IP, *n*=3), and (**B**) intravenous (IV, *n*=4) routes of administration. Absolute quantification qPCR was used to detect SaCas9-AAV vector genome (vg) copies using primers against the SaCas9 transgene and data normalized to the copy number of host genomes (hg) measured using qPCR primers for *Actb*. Mean copy numbers are indicated by horizontal bars, and the mean value shown next to each column.

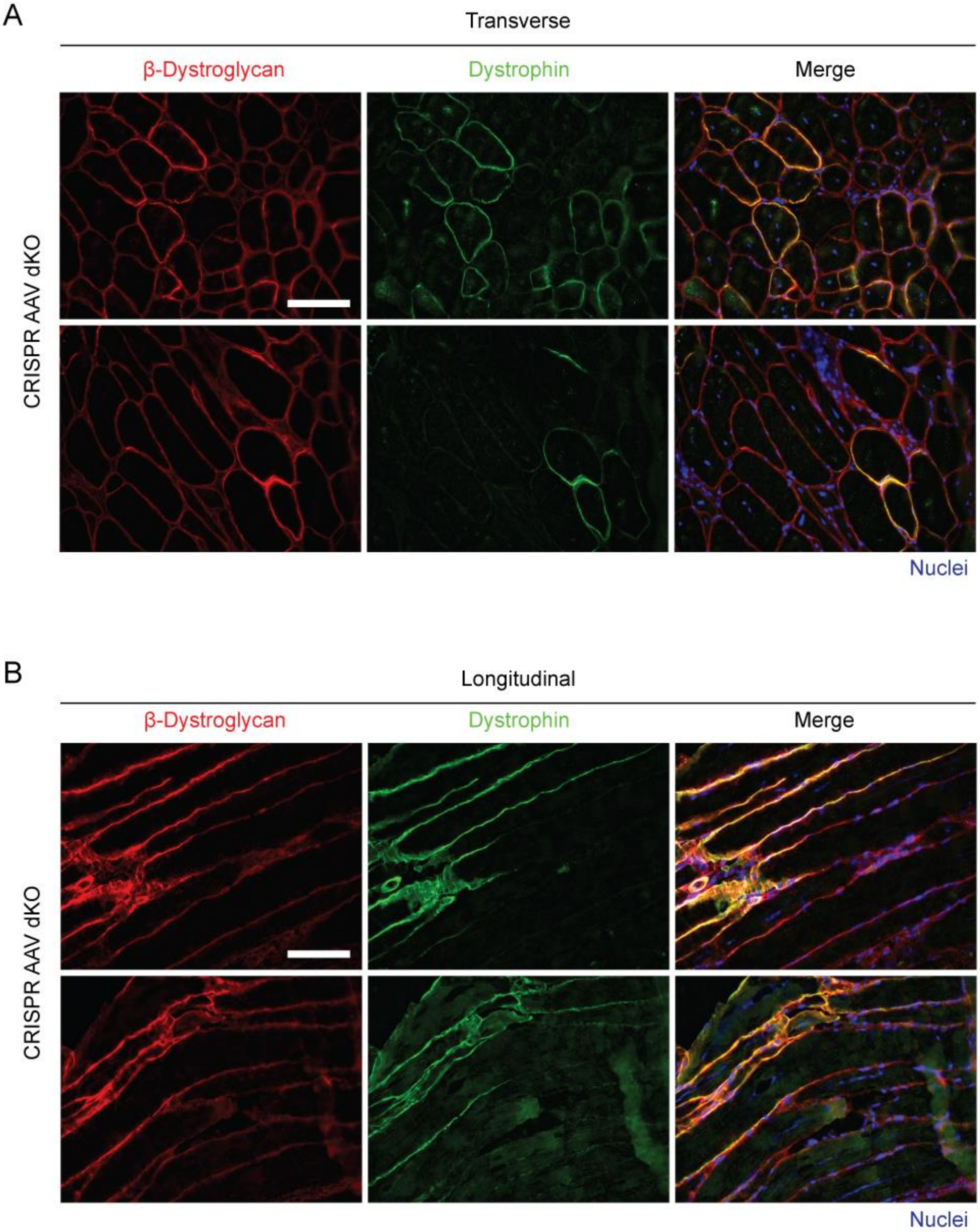
A
Transverse
β-Dystroglycan
Dystrophin
Merge
CRISPR AAV dKO
Nuclei
B
Longitudinal
β-Dystroglycan
Dystrophin
Merge
CRISPR AAV dKO
Nuclei

Figure S4

β-dystroglycan is expressed in a within-fiber patchy manner, and is colocalized with dystrophin in dKO muscle after CRISPR AAV treatment.

Representative β-dystroglycan IF staining in (**A**) transverse and (**B**) longitudinal TA muscle sections of dKO mice treated with CRISPR AAV9 particles. Sections were co-stained for dystrophin and nuclei stained with DAPI. Images were taken at 20× magnification, and scale bars represent 100 μm.

A

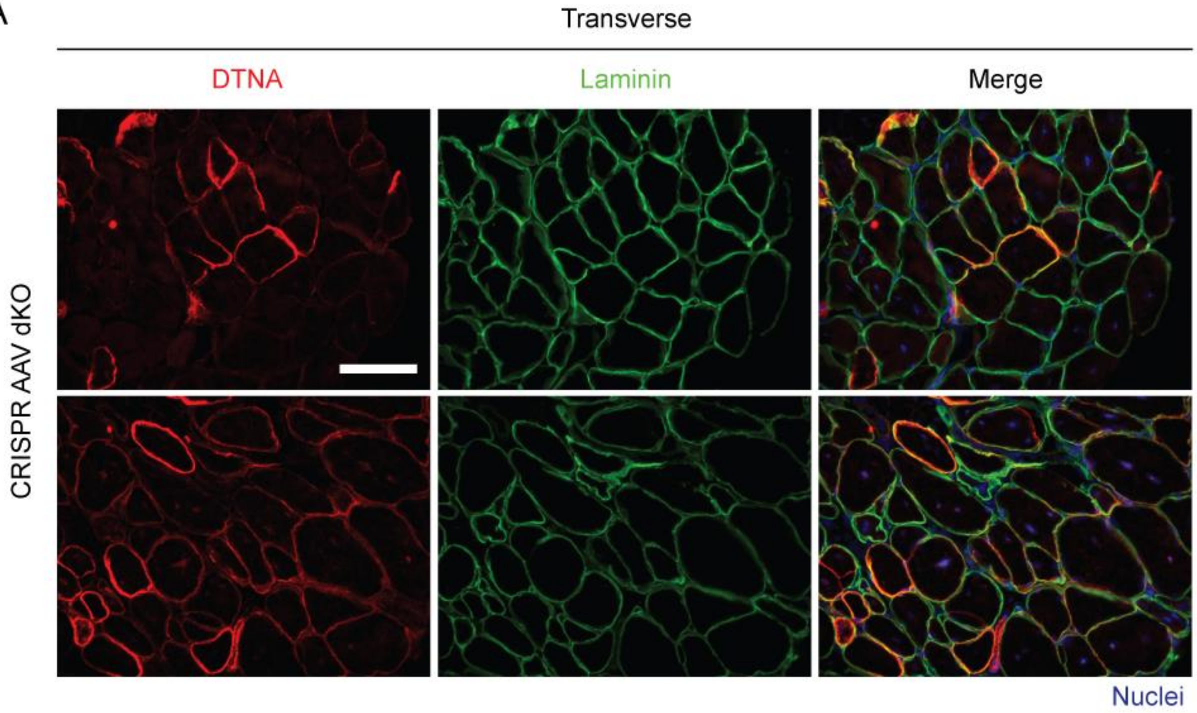
Transverse
DTNA
Laminin
Merge
CRISPR AAV dKO
Nuclei

B

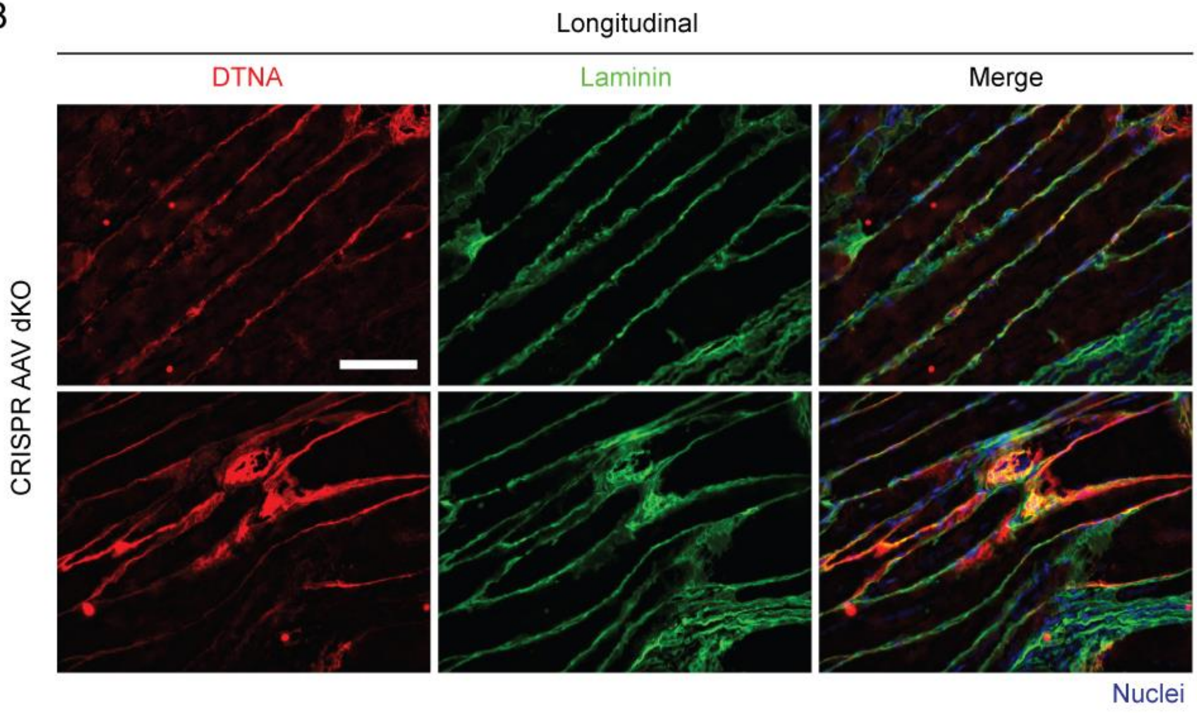
Longitudinal
DTNA
Laminin
Merge
CRISPR AAV dKO
Nuclei

Figure S5

DTNA protein is re-expressed in a within-fiber patchy manner in dKO muscle after CRISPR AAV treatment.

Representative DTNA (α-dystrobrevin) IF staining in (**A**) transverse and (**B**) longitudinal TA muscle sections of dKO mice treated with CRISPR AAV9 particles. Sections were co-stained for laminin to delineate myofiber boundaries and nuclei stained with DAPI. Images were taken at 20× magnification, and scale bars represent 100 μm.

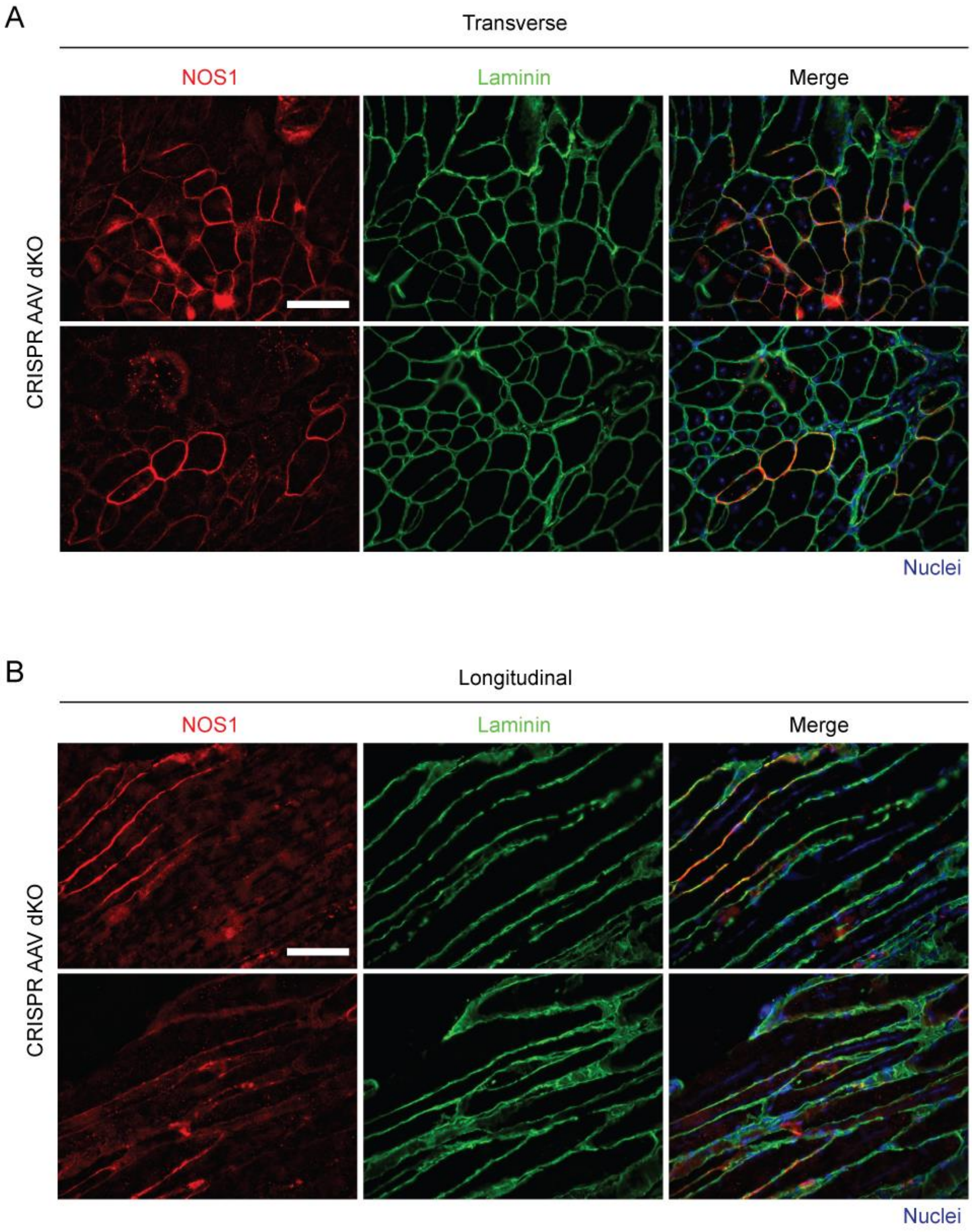
A
Transverse
NOS1
Laminin
Merge
CRISPR AAV dKO
Nuclei
B
Longitudinal
NOS1
Laminin
Merge
CRISPR AAV dKO
Nuclei

Figure S6

NOS1 protein is expressed in a within-fiber patchy manner in dKO muscle after CRISPR AAV treatment.

Representative NOS1 (neuronal nitric oxide synthase, nNOS) IF staining in (**A**) transverse and (**B**) longitudinal TA muscle sections of dKO mice treated with CRISPR AAV9 particles. Sections were co-stained for laminin to delineate myofiber boundaries and nuclei stained with DAPI. Images were taken at 20× magnification, and scale bars represent 100 μm.

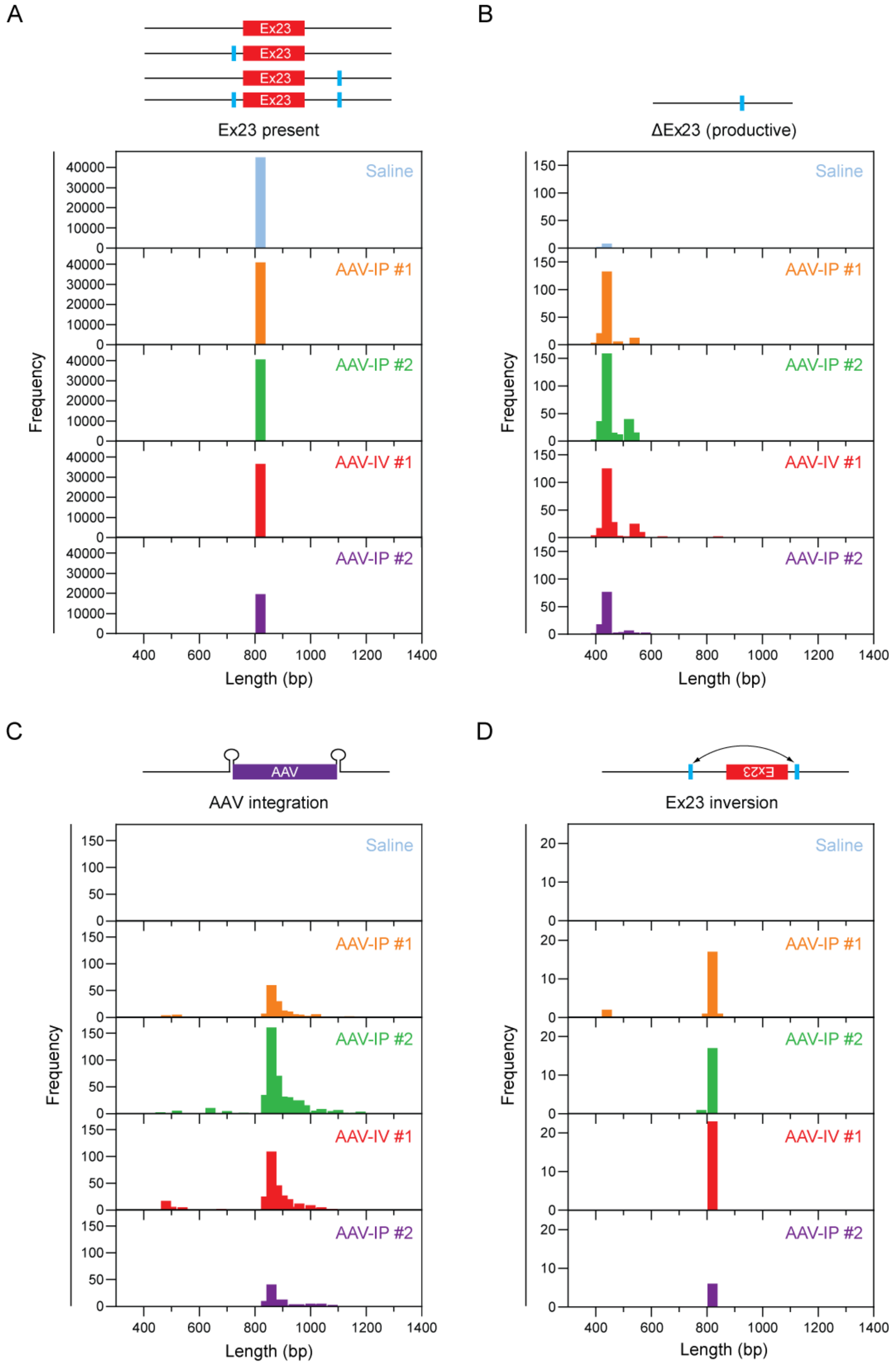
A
Ex23
Ex23 present
Frequency
Saline
AAV-IP #1
AAV-IP #2
AAV-IV #1
AAV-IP #2
Length (bp)
B
ΔEx23 (productive)
C
AAV
AAV integration
D
Ex23
Ex23 inversion

Figure S7

Size distributions for read classification groups following long-read amplicon sequencing. Sequenced amplicons from CRISPR-treated dKO heart samples, or saline control, were assigned to separate categories (**Figure 8**) and read length size distributions determined for (**A**) unedited sequence/indels present at the sgRNA cut site(s), (**B**) ΔEx23 (productive editing), (**C**) presence of integrated AAV-derived sequences, and (**D**) *Dmd* Ex23 present in the reverse orientation (i.e. Ex23 inversion). Schematic representations of possible amplicons are shown for each category.

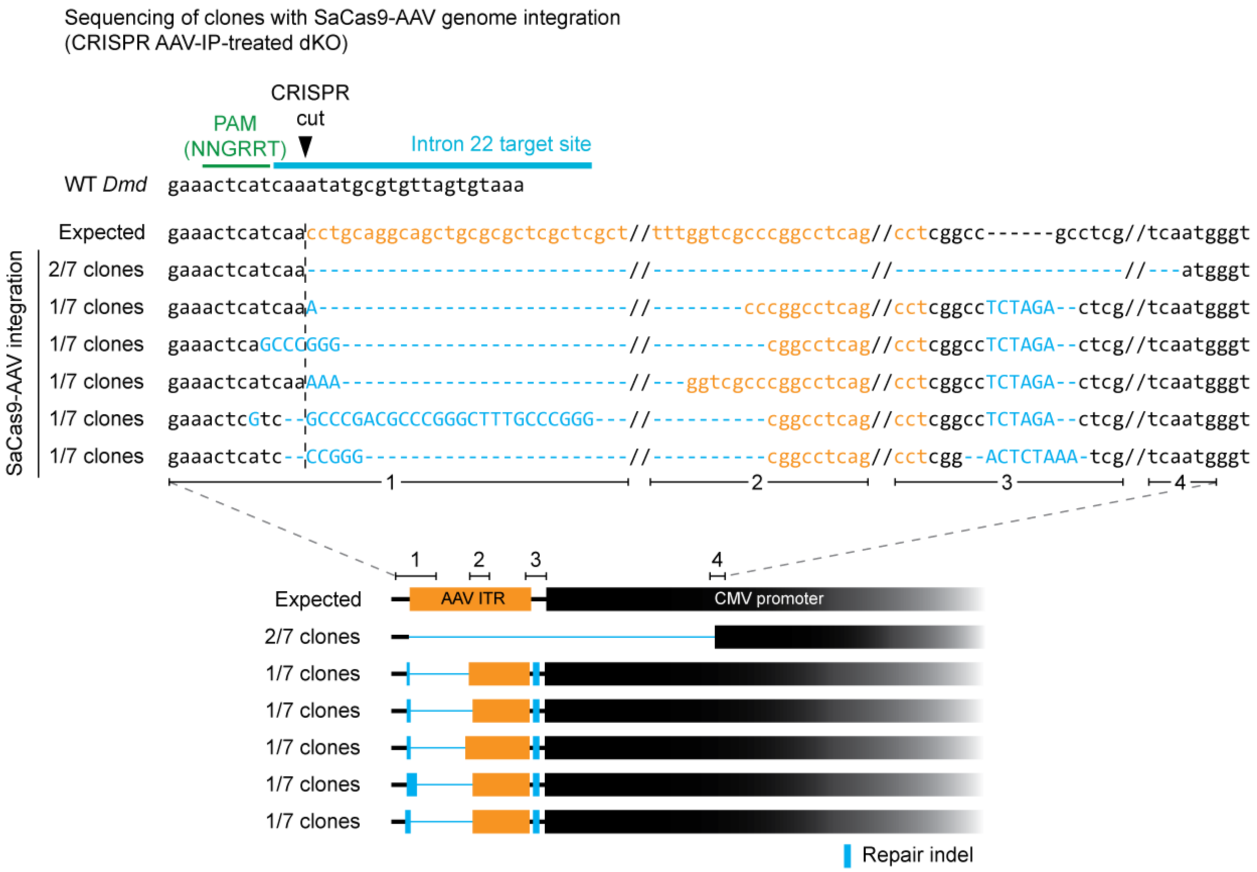

Figure S8

Detection of SaCas9-AAV vector backbone at the 5′ CRISPR cut site by cloning and Sanger sequencing.

PCR was carried out on DNA harvested from the heart tissue of dKO mice treated via intraperitoneal (IP) or intravenous (IV) injection of CRISPR AAVs with primers specific to *Dmd* intron 22 and the CMV promoter of the SaCas9-AAV genome (**Figure 8D**). Amplicons obtained from one AAV-IP treated animal were cloned into shuttle vectors and seven single clones analyzed by Sanger sequencing. Fragments of the SaCas9-AAV vector backbone was detected in all clones tested. Uppercase nucleotides indicate insertions of random bases not present in the WT sequence.
